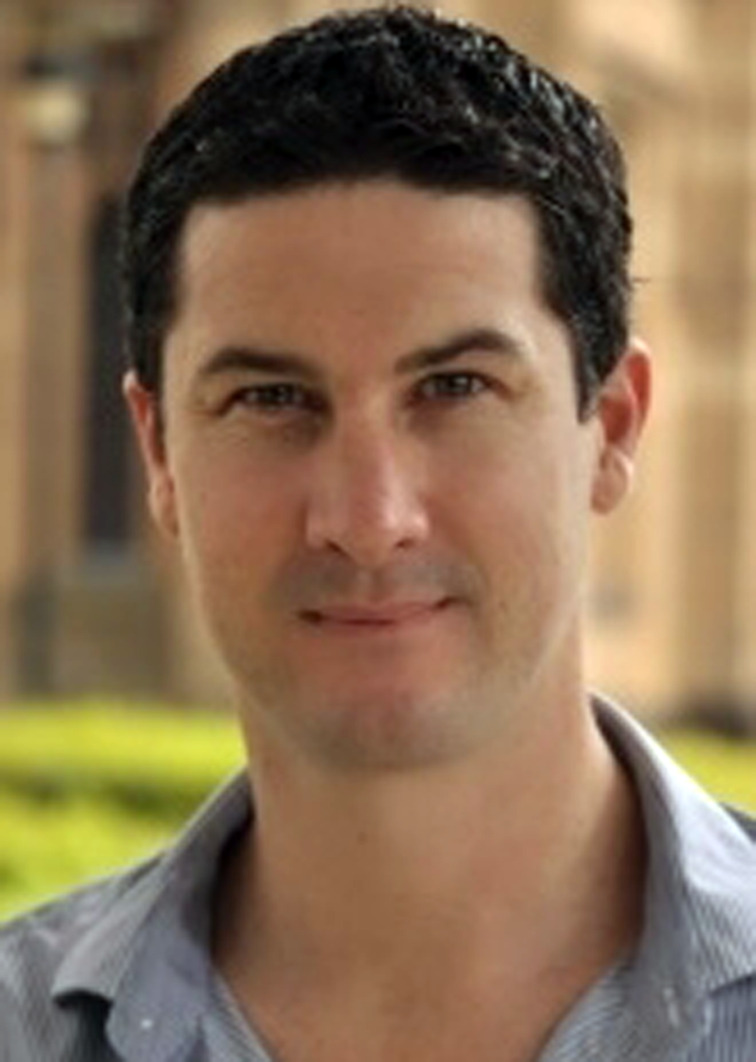# Chemical Synthesis and Semisynthesis of Lipidated Proteins

**DOI:** 10.1002/anie.202111266

**Published:** 2022-02-03

**Authors:** Cameron C. Hanna, Julia Kriegesmann, Luke J. Dowman, Christian F. W. Becker, Richard J. Payne

**Affiliations:** ^1^ School of Chemistry The University of Sydney Sydney NSW 2006 Australia; ^2^ Institute of Biological Chemistry Faculty of Chemistry University of Vienna Vienna Austria; ^3^ Australian Research Council Centre of Excellence for Innovations in Peptide and Protein Science The University of Sydney Sydney NSW 2006 Australia

**Keywords:** chemical protein synthesis, lipidated protein, lipopeptides, post-translational modification, semisynthesis

## Abstract

Lipidation is a ubiquitous modification of peptides and proteins that can occur either co‐ or post‐translationally. An array of different lipid classes can adorn proteins and has been shown to influence a number of crucial biological activities, including the regulation of signaling, cell–cell adhesion events, and the anchoring of proteins to lipid rafts and phospholipid membranes. Whereas nature employs a range of enzymes to install lipid modifications onto proteins, the use of these for the chemoenzymatic generation of lipidated proteins is often inefficient or impractical. An alternative is to harness the power of modern synthetic and semisynthetic technologies to access lipid‐modified proteins in a pure and homogeneously modified form. This Review aims to highlight significant advances in the development of lipidation and ligation chemistry and their implementation in the synthesis and semisynthesis of homogeneous lipidated proteins that have enabled the influence of these modifications on protein structure and function to be uncovered.

## Graphical abstract

Lipidation is a co‐ and post‐translational modification that is known to influence the structure, function, and localization of peptides and proteins. This Review aims to highlight significant advances in the development of modern synthetic and semisynthetic methods for the generation of homogeneously lipidated proteins that have underpinned the study of the effects of lipid modifications on protein structure and function with unprecedented precision.

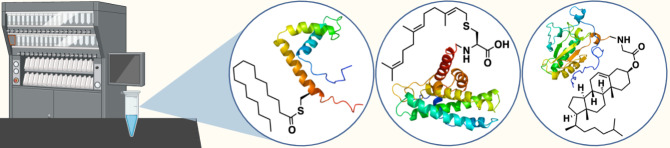

Proteins are responsible for orchestrating the vast majority of biological processes that occur in living systems. Advances in genetics and proteomics have revealed that the total number of proteins within a cell (the proteome) is far larger than the size of the genome; for example, in humans, approximately 20 000 genes are estimated to encode over one million protein products.[^1^, ^2^, ^3^] This enormous diversification of genetic information is largely a result of co‐ and post‐translational modifications (PTMs) that occur during or after protein translation on the ribosome, respectively.[^1^, ^3^] Hundreds of distinct PTMs have been discovered to date and can occur via enzymatic or non‐enzymatic processes.[^4^, ^5^] The nature of these modifications varies from the addition of small functionalities (e.g. phosphorylation, methylation, sulfation, or acetylation), to the addition of larger and/or structurally complex biomolecules (e.g. ubiquitination, glycosylation, ADP‐ribosylation, or lipidation).[^1^, ^6^] Other common PTMs include subtle modifications of amino acid side chains (e.g. citrullination), polypeptide cleavage, and cyclization events.^[5]^ Although there is growing evidence that PTMs occur on a large proportion of human proteins^[7]^ and are crucial for structure, localization, and/or biological function (including the efficacy of many biologics),^[8]^ the modulatory effects of most modifications are unknown for the majority of the proteome.[^2^, ^3^]

Lipidation is a widespread modification of proteins that can occur post‐translationally or co‐translationally. Characterized by the addition of hydrocarbon chains of various lengths to proteins, lipidation increases the protein hydrophobicity, which often leads to membrane anchoring.[^9^, ^10^] This localization at cell (or intracellular) membranes can serve a range of functions, including modulation of the activity of cell‐signaling proteins, sequestration of a protein from a substrate, or the enhancement of protein–substrate association through membrane clustering.[^9^, ^10^] In addition to the wide‐ranging roles of protein‐bound lipids in biology, these molecules have also been implicated in human disease; for example, lipidation of the human oncoprotein, Src, leads to delivery of the protein to the plasma membrane, which results in its pathogenicity.^[11]^ These lipid modifications include prenylation at cysteine (Cys) residues (e.g. *S*‐farnesyl and *S*‐geranylgeranyl lipids), fatty acylation at either Cys residues or the N‐terminus (e.g. *S*‐ and *N*‐palmitoyl or *N*‐myristoyl lipids), and the attachment of cholesterol, glycosylphosphatidylinositol (GPI), or phosphatidylethanolamine (PE) anchors to the C‐terminus (Figure 1). Given the functional importance of protein lipidation, and the diversity of lipid modifications that exist in nature, tools which facilitate access to these biomolecules in homogeneous form are vitally important for detailed structure–function studies.^[12]^ This Review aims to highlight the biological significance of protein lipidation as well as provide a detailed account of the synthetic and semisynthetic technologies that have been developed and employed to efficiently access this class of modified proteins.

**Figure 1:**
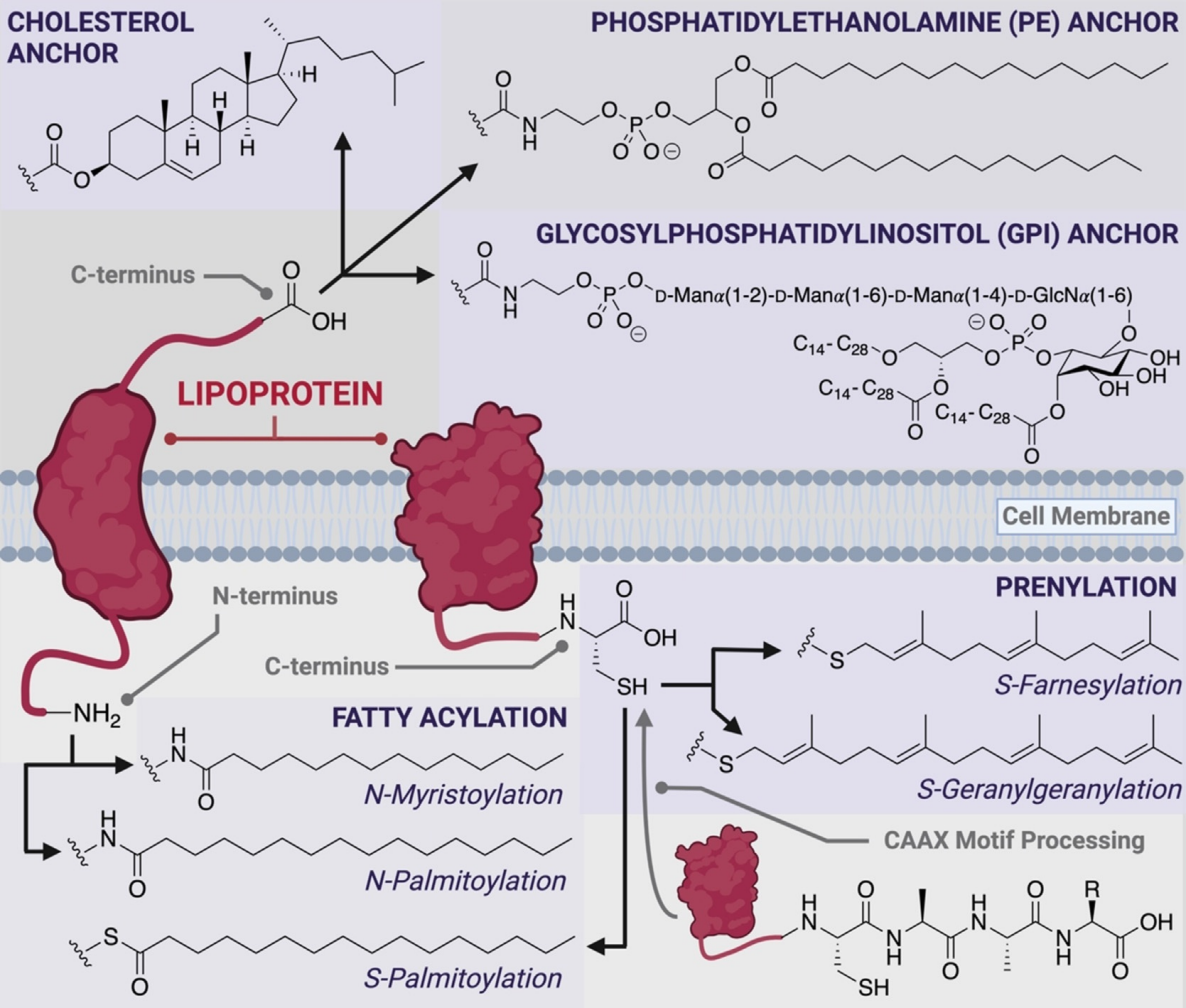
Overview of the different classes of co‐ and post‐translational peptide and protein lipid modifications. Note that prenylation can occur on cytosolic proteins (not shown) in addition to integral membrane proteins.

## 1.1. Prenylation

Prenylation is characterized by the attachment of multiple isoprene units to Cys residues through a thioether linkage within the C‐terminal region of a given protein. The two forms of prenylation are farnesylation and geranylgeranylation, which contain three and four isoprene units, respectively (Figure 1). Up to 2 % of cellular proteins are known to be prenylated in mammalian cells, most of which are geranylgeranylated.^[13]^ Functionally, prenyl modifications serve to recruit otherwise soluble proteins to the cell membrane: this can be either the plasma membrane or endomembranes surrounding organelles such as the Golgi, ER, lysosomes, and nucleus.^[9]^ Examples of proteins that have their function modulated by post‐translational prenylation include the Ras superfamily, which play a central role in cellular signaling. Some commonly farnesylated proteins in this family include K‐Ras, N‐Ras, H‐Ras, Rheb, nuclear lamins, and Hdj2, while geranylgeranylated proteins include Rac, Cdc42, RhoA, and Rab proteins.^[14]^

Until very recently, three protein prenyltransferase enzymes were reported to be operational in eukaryotic cells, being responsible for the installation of modifications to the side chain of Cys residues. Specifically, farnesyl groups are transferred by farnesyltransferase (FTase) using farnesyl pyrophosphate (Fpp) as the substrate, while geranylgeranyl groups are installed by geranylgeranyltransferase (GGTase‐I) using geranylgeranyl pyrophosphate (GGpp).^[9]^ Both of these enzyme‐catalyzed modifications occur within a C‐terminal CAAX box motif, where C is the Cys residue that is modified, A is an aliphatic amino acid, and the nature of the X residue dictates the type of modification installed. For example, when X is serine (Ser), methionine (Met), or glutamine (Gln), the Cys residue is farnesylated, whereas when X is leucine (Leu), the protein is geranylgeranylated.[^15^, ^16^] There is also evidence to suggest that FTase strongly prefers small hydrophobic residues present at the second A position.^[17]^ After prenylation, the remaining amino acids (AAX) of the box are cleaved, either by an endoplasmic reticulum protease or Ras‐converting enzyme 1. The resulting carboxylate group of the side‐chain‐prenylated Cys residue is subsequently methylated by the enzyme isoprenylcysteine carboxymethyltransferase (ICMT) to provide a C‐terminal methyl ester.^[18]^ In contrast to the FTase and GGTase‐I prenyltransferases, the third class of enzyme, Rab (Ras‐related in brain) geranylgeranyl transferase (GGTase‐II or Rab‐GGTase) employs GGpp as a substrate to specifically transfer either one or two geranylgeranyl groups. The C‐terminal prenylation motifs that are found within the family of Rab proteins are mostly CC and CXC but also include CCX, CCXX, and CXX.[^9^, ^19^] A fourth type of protein prenyltransferase called GGTase3 has very recently been discovered and is responsible for geranylgeranylating the ubiquitin ligase FBXL2, thereby linking it to the membrane and allowing polyubiquitylation of membrane‐anchored proteins.^[20]^ It similarly modifies SNARE proteins, such as Ykt6, which is a prerequisite for proper assembly of the Golgi SNARE complex.^[21]^

## 1.2. Fatty Acylation

Myristoylation and palmitoylation represent the two most common forms of protein fatty acylation, and both have been shown to critically influence protein structure, function, and/or localization.[^9^, ^22^, ^23^] Protein palmitoylation is defined by the attachment of a C_16_ palmitate fatty acid to a protein and can occur through two different linkages in humans (Figure 1). In S‐palmitoylation, the lipid is reversibly attached to the side chain of Cys residues through an enzymatically and hydrolytically labile thioester linkage. In contrast, with N‐palmitoylation, the lipid is transferred to the N‐terminus of the protein or, more rarely, to a lysine side chain where it is appended through a hydrolytically stable amide bond. Interestingly, no enzymatic machinery is known to remove N‐palmitoylation and it is, therefore, thought to be an irreversible modification.^[22]^ Unlike many other PTMs, including prenylation, a specific consensus sequence for predicting protein N‐palmitoylation does not exist and, in addition, some reported N‐palmitoylation modifications could be the result of S to N transfer reactions.^[24]^ S‐palmitoylation, however, is often associated with nearby N‐myristoylated glycine (Gly) residues, or prenylated C‐terminal Cys residues.^[22]^

Palmitoylation is implicated in protein trafficking, as the imparted hydrophobicity directs the otherwise soluble proteins to different cellular and organelle membranes. In neurons, palmitoylation is important for targeting proteins to the axon terminals, which ultimately regulates synapse activity.^[25]^ For many proteins, S‐palmitoylation is not permanent but rather cycles between palmitoylation and depalmitoylation to regulate their function in a dynamic manner.^[26]^ Such dynamic modification cycles are driven enzymatically, with addition of the lipid being carried out by palmitoyltransferases and removal being catalyzed by acylprotein thioesterases, such as acylprotein thioesterase‐1 (APT1)^[27]^ or palmitoylthioesterase‐1 (PPT1).^[28]^ Members of the Ras protein family of small GTPases are some of the most well‐studied examples of S‐palmitoylated proteins. Here, the lipidation has been shown to regulate the membrane association of the proteins.^[29]^ There is also evidence to suggest that palmitoylation protects proteins from proteasomal degradation by preventing their ubiquitination.[^30^, ^31^]

Protein N‐myristoylation is defined by the attachment of a C_14_ myristoyl group to an N‐terminal Gly residue of a protein through an amide linkage. In some cases, proteins can also bear myristoyl groups at the ϵ‐amino group of lysine (Lys) residues, as is the case for TNFα and the precursor interleukin 1α protein.[^32^, ^33^, ^34^] Unlike prenylation and palmitoylation, myristoylation can occur co‐translationally as well as post‐translationally, with the myristoyl group installed after cleavage of the N‐terminal initiator Met residue by methionine aminopeptidase.^[35]^ In eukaryotes, *N*‐myristoyltransferases (NMT1 or NMT2) are primarily responsible for catalyzing the transfer of the myristoyl group from myristoyl‐CoA to the substrate protein bearing an N‐terminal Gly residue.^[36]^ Like other forms of lipidation, N‐myristoylation regulates cell signaling, membrane association, and trafficking, and dual myristoyl modifications are also common.[^37^, ^38^] In many cases, a single myristoyl group is not sufficient to induce membrane trafficking, as additional lipidation is necessary to enhance hydrophobicity. For this reason, myristoylation and palmitoylation are commonly found together on proteins.^[39]^

It is important to note that rare protein fatty acylations also occur with octanoate (best known as *O*‐octanoate modification on the peptide hormone ghrelin),^[40]^ as well as with unsaturated C_16_–C_20_ fatty acid chains.

## 1.3. Glycosylphosphatidylinositol Anchors

The attachment of a glycosylphosphatidylinositol (GPI) anchor (also called glypiation) is a post‐translational modification found across a broad range of organisms, including mammals, insects, plants, fungi, and protozoa.^[41]^ The modification occurs on the C‐terminus of proteins and typically serves to anchor proteins to the extracellular face of plasma membranes.^[41]^ The GPI structure comprises a phosphoethanolamine linker, a highly conserved glycan core (d‐Man(α1‐2)‐d‐Man(α1‐6)‐d‐Man(α1‐4)‐d‐GlcN(α1‐6)*myo*‐inositol), and a lipid tail which varies in structure depending on the organism it originates from. Specifically, these lipids can vary in length from 14 to 28 carbon atoms, and can either be saturated or unsaturated.^[42]^ Although many of the biological functions of the GPI anchor are yet to be elucidated, they have already been shown to play key roles in cell–cell adhesion, signal transduction, membrane targeting, and lipid raft partitioning.[^43^, ^44^, ^45^] Bertozzi and co‐workers have undertaken numerous elegant chemical biology studies to understand the structure–function relationships of GPI anchors. For example, work from the group has shown that the internal glycan of the GPI is important for lateral mobility of proteins to regulate activity.[^46^, ^47^] Using a powerful cell surface painting strategy, purified GPI‐modified proteins have been anchored into cell membranes from exogenous sources in both in vitro and in vivo settings.[^48^, ^49^, ^50^] GPI anchors have also been shown to be important components of immunodominant epitopes for eukaryotic parasites (e.g. *Plasmodium falciparum*), which has encouraged the production of synthetic variants for use as vaccine candidates.[^51^, ^52^] Crucial to these studies is the ability to access sufficient quantities of GPI‐anchored peptide or proteins in pure form. When produced through recombinant expression in cells, samples are typically heterogeneous, with a wide variety of structures within the lipid portion of the GPI that are very challenging to separate by chromatographic techniques.[^45^, ^53^] As such, chemical synthesis has emerged as a potential avenue to access GPI‐anchored proteins for functional studies. To avoid the challenging and labor‐intensive synthesis of the native GPI molecule, several simplified mimics have been synthesized, studied, and reviewed; however, these will not be discussed in detail in this Review.[^45^, ^54^, ^55^]

## 1.4. Cholesterol Anchors

Cholesterol anchors are found in the context of hedgehog (Hh) proteins, which are important for embryonic development and malignant tumorigenesis in a variety of tumor types.[^56^, ^57^] These cholesterol modifications are incorporated during a unique autocleavage process, after which the 3β‐hydroxy group of cholesterol is linked to the C‐terminus of the processed protein through an ester linkage (Figure 1).[^56^, ^57^] This reaction is initiated by intramolecular nucleophilic attack on the carbonyl group of a Gly residue by an adjacent thiol side chain of a Cys residue, leading to the intermediate formation of a thioester linkage. This thioester subsequently reacts with the 3β‐hydroxy group of cholesterol to generate an ester linkage and liberate the C‐terminal autoprocessing domain.^[9]^ C‐terminal cholesterol anchors have been shown to be responsible for the release of dually lipidated Hh proteins from the cell surface. This is facilitated by two transporter‐like proteins (Scube and Disp) that recognize parts of the cholesterol molecule.[^58^, ^59^, ^60^] Importantly, although cholesterol is not necessarily required for Hh signaling activity, it has been shown that the absence of this modification reduces signaling potency.^[59]^ Modification with cholesterol has also been shown to be important in regulating the activity of the protein smoothened (SMO), which is modified on an aspartic acid (Asp) residue rather than the C‐terminus of the protein.^[61]^

## 1.5. Phosphatidylethanolamine Anchors

The addition of phosphatidylethanolamine (PE) modifications to generate PE anchors is a rare and relatively understudied PTM. To date, PE anchors have been found on the autophagy‐related proteins Atg8 (yeast) and LC3 (mammals).^[62]^ These anchors are covalently attached to proteins through an amide bond between a C‐terminal Gly residue and the amino group of the PE.^[63]^ In both the Atg8 and LC3 proteins, the conjugation to PE is essential for their correct localization and function.^[64]^

## 2. Tools for the Preparation of Lipidated Peptides and Proteins

### 2.1. Synthetic Tools

**Figure 2:**
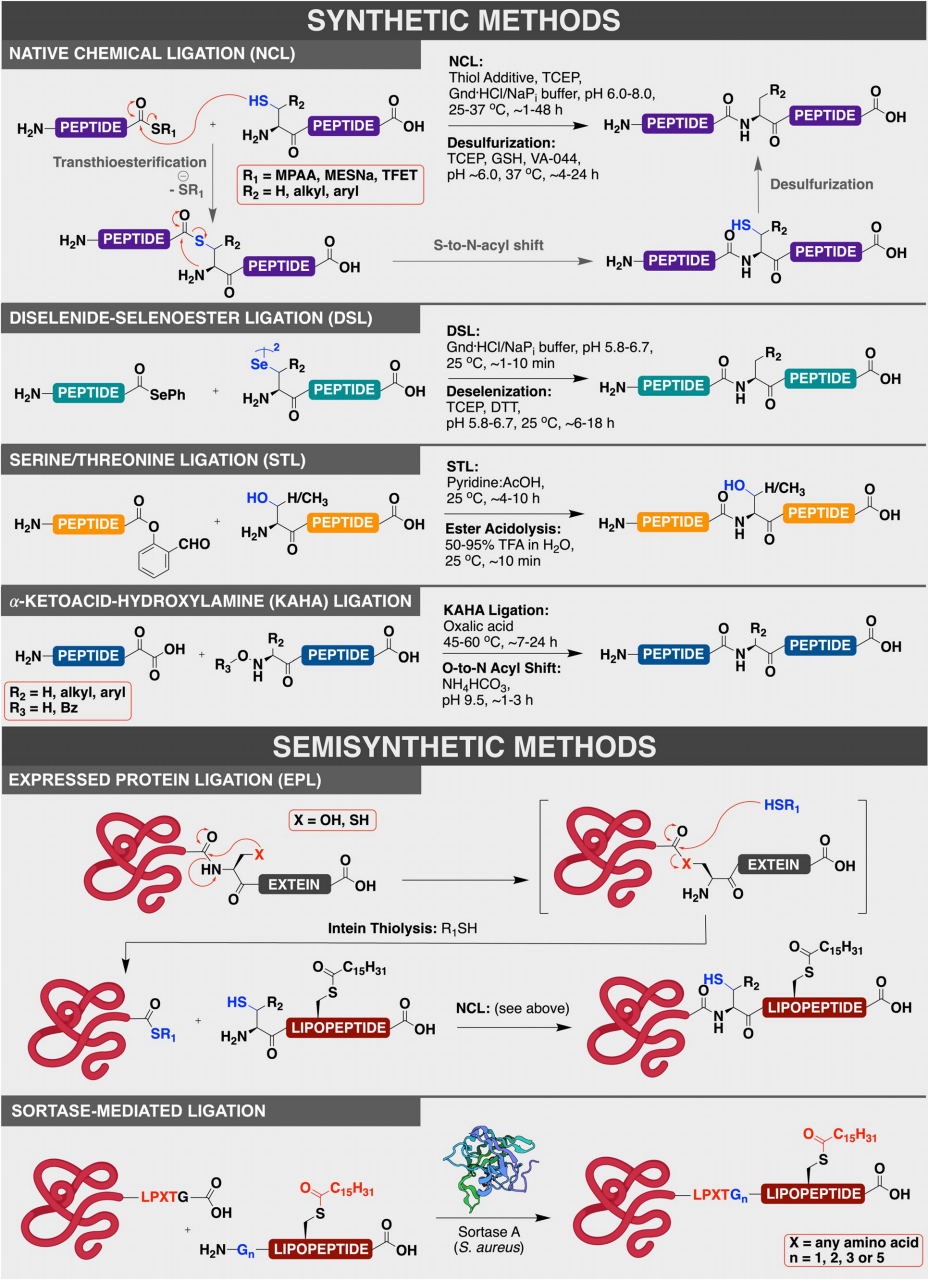
General overview of the different strategies used to access lipidated proteins by chemical synthesis or semisynthesis.

In general, lipopeptides can be routinely accessed by standard solid‐phase peptide synthesis (SPPS) techniques. Although several solution‐phase syntheses of lipopeptides have been reported,[^65^, ^66^, ^67^, ^68^] these approaches tend to be laborious and require numerous protecting group manipulation and purification steps. Moreover, the inherent solubility problems that accompany the use of side‐chain‐protected peptides in solution‐phase peptide synthesis are exacerbated for lipopeptides.^[64]^ These issues are mitigated on a solid support as reactions can be driven to completion with excess reagents, which can be removed by simple filtration. Lipopeptides can be accessed by SPPS either by coupling pre‐lipidated amino acids to a growing chain, or by selectively lipidating specific unprotected amino acids following complete elongation of the chain.^[69]^ The production of full‐length lipid‐modified proteins is considerably more challenging; this is due to the inherent size limits of peptides that can be assembled by standard SPPS methods (typically 40–50 residues). For these reasons, lipidated peptides and proteins of more than 50 amino acids in length are more commonly accessed by peptide ligation chemistry, in which a peptide and lipopeptide fragment can be chemoselectively fused to access larger targets (Figure 2). Larger lipidated protein targets that would be intractable or impractical to produce through total chemical synthesis can instead be generated through semisynthetic methods. As a methodology, protein semisynthesis is broadly categorized as the use of either chemical or chemoenzymatic methods to fuse a synthetic peptide to a larger and (typically) unmodified expressed protein (Figure 2). One of the most widely adopted methods for protein semisynthesis is expressed protein ligation (EPL)—which leverages the NCL manifold to facilitate chemical ligation of a recombinant protein with a synthetic peptide. This was first demonstrated through ligation of a recombinant protein thioester (generated through thiolysis of a protein–intein fusion) and a synthetic peptide bearing an N‐terminal Cys residue. Alternatively, a number of methods have been developed for chemoenzymatic semisynthesis, the most relevant to this Review being the sortase‐mediated ligation, whereby a protein tagged with a C‐terminal sortase‐recognition sequence can be regioselectively fused to a C‐terminal peptide or protein by the sortase enzyme (Figure 2).

Recombinant expression methods for accessing proteins are routine and most commonly performed in *E. coli*.[^70^, ^71^] In addition to their ease of genetic manipulation and handling, *E. coli* grows rapidly and typically provides high yields of a target protein. Nonetheless, some complex mammalian proteins that feature high degrees of structural complexity can be challenging to access in simple bacterial expression systems. Furthermore, although prokaryotes are known to post‐translationally modify proteins, the use of *E. coli* expression systems mostly results in the production of unmodified proteins, and it is not straightforward to use standard bacterial systems for the incorporation of mammalian PTMs.^[71]^ It should be noted that eukaryotic expression systems such as yeast^[72]^ or insect cells^[73]^ bear the necessary enzymatic machinery to generate many higher‐order PTMs and have been used to access modified proteins. Such approaches, however, are intrinsically plagued by the formation of heterogeneous mixtures of differentially modified proteins. This severely limits their application for deconvoluting the effects of particular PTMs and for accessing more highly defined, site‐specifically modified protein therapeutics, a class of biomolecules predicted to form the bedrock of the burgeoning biotechnology and “biologics” industries.[^64^, ^74^] For this reason, the semisynthetic generation of lipidated proteins through the chemoselective ligation of an unmodified recombinant protein and a homogeneously lipidated synthetic peptide represents an enormously powerful methodological platform.

Assembly of full‐length lipid‐modified proteins from segments produced synthetically or recombinantly can be achieved through a range of diverse ligation techniques (Figure 2).[^3^, ^75^, ^76^] The most commonly used method is native chemical ligation (NCL);[^77^, ^78^] however, other common ligation methods include the diselenide‐selenoester ligation (DSL),[^79^, ^80^, ^81^] Ser/Thr ligation (STL),^[82]^ α‐ketoacid‐hydroxylamine (KAHA) ligation,^[83]^ maleimidocaproyl (MIC) ligation,^[68]^ and sortase‐mediated enzymatic ligation.^[84]^ In most cases, unprotected peptide and protein fragments are used, which allows reactions to be performed in buffered aqueous solutions at neutral (or near‐neutral) pH and ambient‐to‐moderate temperatures. The NCL method involves a chemoselective reaction between a peptide bearing an N‐terminal Cys residue with another peptide derivatized as a C‐terminal thioester. Mechanistically, the NCL reaction is initiated by nucleophilic attack of the side chain of the Cys residue (at the N‐terminus of one segment) at a thioester (at the C‐terminus of the other segment) in a reversible transthioesterification step. This step is followed by a rapid S‐to‐N‐acyl shift to produce the native amide bond.[^77^, ^78^] The synthesis of peptide thioesters can be achieved using a range of solution‐ and solid‐phase procedures.^[85]^ Larger protein thioesters can also be accessed using engineered inteins, which utilize a natural protein splicing process.^[86]^ In this process, an internal peptide fragment within a protein, termed an intein, is self‐excised from the larger protein, which then ligates two flanking segments, termed exteins, thereby forming an amide bond between them. In the first step, a (thio)ester is formed at an N‐terminal Cys or Ser residue of the intein by a reversible *N*‐to‐*S/O*‐acyl shift (Figure 2). This intermediate is then subjected to a trans(thio)esterification after nucleophilic attack by a Cys, Ser, or Thr residue present on the C‐terminus of the extein. The resulting (thio)ester then undergoes an intramolecular cyclization at the conserved asparagine (Asn) residue present on the C‐terminus of the intein. This succinimide formation excises the intein and after a final *S*/*O*‐to‐*N* acyl shift, this ultimately results in the formation of an amide bond between the two exteins.[^87^, ^88^] In the context of EPL, a fusion construct of the target protein linked to an intein domain that can only undergo the initial thioester formation is employed, and affinity tags such as chitin‐binding domains (CBDs) can be incorporated on the C‐terminus of this construct to facilitate downstream protein purification. After purification by affinity chromatography, the fusion protein can be cleaved and eluted from the column with an excess of a thiol such as 2‐mercaptoethanesulfonate (MESNa), thereby providing the corresponding MESNa thioester of the desired protein segment. This can then be ligated to a synthetic lipopeptide containing an N‐terminal Cys by an NCL reaction.[^64^, ^87^]

The DSL methodology is inspired by the NCL reaction but harnesses the superior reactivity of C‐terminal selenoesters with the enhanced nucleophilicity of the 21st amino acid selenocysteine (Sec) on the N‐terminus of the other peptide fragment. This enhanced reactivity means that ligation reactions are more facile than NCL and can be performed even at low concentrations. DSL is, therefore, a useful ligation technique when reaction partners are less soluble (e.g. for lipidated fragments), and has been successfully employed for lipidated protein synthesis.^[89]^ STL involves the chemoselective reaction between an unprotected peptide with a C‐terminal salicylaldehyde (SAL) ester and another unprotected peptide with an N‐terminal Ser or threonine (Thr) residue.[^90^, ^91^] The high abundance of Ser and Thr residues in native proteins makes this an attractive method for protein synthesis. A final type of peptide ligation reaction covered here is the α‐ketoacid‐hydroxylamine (KAHA) ligation.[^83^, ^92^] This reaction proceeds via the decarboxylative condensation of a peptide bearing an α‐ketoacid on the C‐terminus with a peptide containing an N‐terminal hydroxylamine functionality. This enables the fusion of the fragments through an amide bond, typically under acidic organic buffering conditions. Despite the availability of these key techniques, it is anticipated that future innovations based around these peptide ligation concepts will undoubtedly enhance the number of lipidated protein targets that can be accessed by synthesis and semisynthesis.

Two further methods that have been used to generate lipid‐modified protein analogues are the maleimidocaproyl (MIC) and sortase ligations. Specifically, in the MIC ligation, the synthetic lipopeptide is equipped with an N‐terminal maleimide group;^[68]^ this is reacted with a Cys residue within an expressed protein fragment through Michael addition to the side chain thiol. One benefit of this method is that Cys residues on the C‐terminus can be modified with a degree of selectivity due to the steric inaccessibility of other Cys residues in the sequence if they are buried within the protein.^[93]^ However, this method is not compatible with multiple C‐terminal Lys residues, as the ϵ‐amine functionality can also react with the electrophilic maleimide moiety.^[94]^ In contrast, the sortase ligation capitalizes on the ability of the sortase enzyme to specifically recognize an LPXTG pentapeptide motif [X=preferably Ser or glutamate (Glu)] for chemoselective fusion of two peptide or protein fragments (Figure 2).^[95]^ Following binding to the motif, sortase first initiates thiolysis of the T−G amide bond to produce a thioester‐linked acyl enzyme intermediate. This thioester is then intercepted by the α‐amine moiety of an N‐terminal Gly residue on another peptide or protein fragment to afford the transpeptidation product with regeneration of the active sortase enzyme.[^84^, ^95^] A major drawback of these strategies is that they generate non‐native “scars” within the protein sequence, namely, an unnatural maleimide or LPXTG motif for the MIC and sortase ligations, respectively. Other examples of ligation or bioconjugation techniques that have been successfully employed to access unnatural analogues or mimics of lipidated proteins include the Diels–Alder ligation^[96]^ and the the Cu^I^‐catalyzed azide‐alkyne cycloaddition (CuAAC)— the archetypal click reaction.^[97]^

### 2.2. Tools for Improving the Handling and for Analyzing Lipidated Peptides and Proteins

The introduction of lipid moieties into peptides and proteins can dramatically change their physicochemical properties, leading to poor solubility, and in turn aggregation or the formation of micelles in aqueous buffers.^[98]^ Many efforts have been made to tune the solubility of lipidated peptides and proteins by using buffer additives such as detergents and/or chaotropes, or through the introduction of transient solubility tags. The improved solubility engendered through these strategies aids both ligation and lipid modification reactions, as well as with subsequent handling during purification.

In most cases, lipidated peptides and proteins are purified and analyzed by reverse‐phase high‐performance liquid chromatography (RP‐HPLC), as their increased hydrophobicity allows easy separation from unmodified and/or much smaller precursors. Here, the use of short‐chain stationary phases with sufficiently large pore size is recommended, for example, a C_4_ stationary phase with a pore size of 30 nm. The use of columns with longer alkyl chains such as C_18_ complicate purification because of the higher affinity of the lipid modification to these stationary phases.^[99]^ The choice of organic eluent and modifier for RP‐HPLC is also critical, as is the temperature. Notably, optimized purification conditions usually need to be identified for each protein individually (or at least for a class of proteins) and one must ensure that the use of low pH and/or high temperatures during purification does not lead to decomposition of the proteins or cleavage of lipid chains.^[100]^

In many cases, the addition of organic solvents miscible with water, such as acetonitrile or fluorinated alcohols, help to solubilize lipidated peptides and proteins before and during purification. Fluorinated alcohols such as 2,2,2‐trifluoroethanol (TFE) or 1,1,1,3,3,3‐hexafluoro‐2‐propanol (HFIP) are strong hydrogen bond donors and can stabilize α‐helical secondary structures, thereby helping to keep peptides and proteins with helical structural elements in solution. Combinations of organic solvents (e.g. acetonitrile with 2‐propanol and TFE) with low concentrations of TFA as a modifying agent have been successfully used for the purification of lipidated proteins by RP‐HPLC.^[101]^

Although there are even better solvents for dissolving hydrophobic peptides and proteins such as dimethylformamide (DMF) and dimethyl sulfoxide (DMSO), these are not commonly used due to their strong UV absorption at 214 nm (similar to absorption of the peptide bond), which interferes with the absorption of peptides and proteins during HPLC purification.^[102]^ The addition of DMSO can also lead to the oxidation of methionine and cysteine side chains.^[103]^ If no conditions for RP‐HPLC can be identified, hydrophobic interaction chromatography (HIC) can serve as an alternative means of purification. For this method, a hydrophobic stationary phase (such as agarose with butyl or phenyl ligands) is used and the samples are applied to the column in a high‐salt buffer. A decreasing salt gradient is used to elute proteins from the column in order of increasing hydrophobicity.^[104]^ It should be noted that HIC is not suitable for the purification of peptides and proteins that form aggregates through solely hydrophobic interactions.

In such cases, aqueous buffers containing high concentrations of chaotropes such as guanidinium hydrochloride (Gdn⋅HCl, up to 6 M) or urea (up to 8 M) are often used in combination with detergents to prevent the aggregation of hydrophobic peptides and proteins by disrupting hydrogen bond donor and acceptor sites and by blocking hydrophobic interaction sites with detergent molecules.[^105^, ^106^] Above the critical micelle concentration (CMC), detergents provide an ideal environment for lipids; however, they can also denature proteins and are often challenging to remove by standard purification approaches. The most commonly used detergents include the negatively charged sodium dodecyl sulfate (SDS) as well as non‐ionic detergents such as Triton X‐100 and Tween 20. It is important to note that the polyethylene glycol moieties in Triton X‐100 and Tween 20 are easily ionized and, therefore, suppress other molecules during analysis by mass spectrometry. For this reason, alternative detergents such as octylglycoside (OG) and *n*‐dodecyl‐β‐D‐maltoside (DDM) are often preferred, as they can be removed by dialysis or with the use of biobeads.^[102]^

In cases where the strategies described above (or combinations thereof) do not lead to successful ligation and/or purification, an alternative viable strategy is the covalent attachment of solubility tags that can be removed following the ligation or folding step. Such transiently attached solubilizing molecules complicate synthesis and lead to decreased yields as a result of additional steps, but can significantly improve the recovery of hydrophobic peptides and proteins through RP‐HPLC. One of the earliest reported strategies involved the introduction of polylysine tags (four or more lysine residues) on the N‐ or C‐terminus of transmembrane peptides, and has been successfully used to increase the solubility of peptides derived from the human erythrocyte protein glycophorin A, bacteriophage M13 major coat protein and the hepatitis C virus membrane protein NS4A in aqueous buffers.^[107]^ However, these early versions of solubility tags were permanently attached and, owing to their high charge, can influence the biological function of the peptides and proteins. As a result, temporary solubilization tags such as polyethylene glycol polyamide, polyethylene glycol, and polyarginine moieties were developed. These can be attached on the termini, to side chains, or to the polypeptide backbone through a variety of different linkers that allow cleavage of the tags under acidic^[108]^ or basic^[109]^ conditions or with the use of specific protease enzymes.[^110^, ^111^] Solubility tags linked through photocleavable linkers have also been developed that avoid exposure to harsh reaction conditions during cleavage.^[112]^ Based on the summary provided above, although there are clearly a number of strategies available to handle and purify lipidated peptides and proteins, there is no singular generalizable strategy and the practitioner may in some cases need to test a number of these approaches. Overall, it is recommended that if no solubilizing buffer can be found and other alternatives, such as the use of chaperones (see Section 6 for lipidated Rab proteins), are not available, solubility tags on the backbone of the target peptide/protein are a good option for accessing homogeneously lipidated proteins.

## 3. Synthesis and Semisynthesis of Palmitoylated Peptides and Proteins

**Scheme 1:**
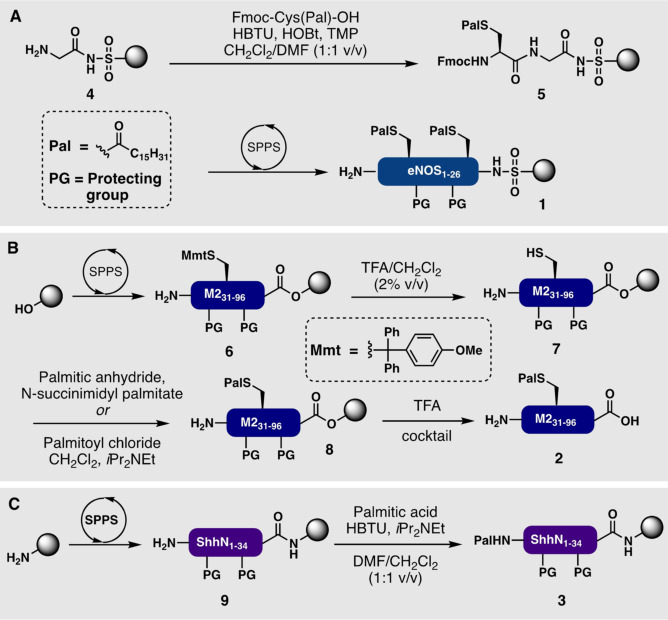
Solid‐phase synthesis of S‐palmitoylated peptides by A) coupling of pre‐lipidated amino acids^[113]^ or B) direct on‐resin palmitoylation of unprotected Cys residues.^[114]^ C) Solid‐phase synthesis of N‐palmitoylated peptides by direct coupling of palmitic acid to the N‐terminus.^[115]^ PG=Standard side‐chain protecting groups employed in Fmoc‐SPPS.

S‐Palmitoylated lipopeptides are typically accessed by SPPS using the Fmoc strategy (with adjusted Fmoc removal conditions to avoid thioester hydrolysis and S‐ to N‐acyl transfer) using pre‐lipidated amino acid building blocks. An example of this strategy was in the synthesis of resin‐bound palmitoylated endothelial nitric oxide synthase (eNOS)_1–26_ (**1**) by Waldmann and co‐workers (Scheme 1 A).^[113]^ An alternative approach involves the introduction of the lipid modification to a substrate peptide at a late stage, as exemplified by the synthesis of a palmitoylated variant of the matrix protein M2_31–96_ (**2**; Scheme 1 B).^[114]^ N‐Palmitoylated lipopeptides are relatively straightforward to prepare through direct condensation of palmitic acid to the N‐terminus of a solid‐supported peptide using standard coupling conditions, as demonstrated in the assembly of the sonic hedgehog N‐terminal fragment (ShhN)_1–34_ (**3**; Scheme 1 C).^[115]^ A common issue encountered with the solid‐phase synthesis of S‐palmitoylated peptides and proteins is that palmitoyl thioesters are labile to standard Fmoc‐deprotection conditions (e.g. 20 vol % piperidine in DMF) and can undergo *S*‐to‐*N* acyl shifts when present on deprotected Cys residues located at the N‐terminus.^[116]^ To prevent this unwanted *S*‐to‐*N* acyl shift, an Fmoc deprotection solution containing 1 vol % DBU in DMF can be used during peptide synthesis, followed immediately by the next amino acid coupling step.^[116]^ A common issue observed during the synthesis of peptides bearing fatty acyl modifications is poor solubility or aggregation leading to the generation of higher order structures. This issue is compounded by the fact that the amphipathic nature of lipopeptides leads to broader elution profiles on stationary phases used for chromatographic purification (e.g. HPLC), thus making it more difficult to remove by‐products during purification steps. For this reason, access to full‐length lipidated proteins usually necessitates the assembly of multiple peptide and lipopeptide fragments using peptide ligation chemistry, or the use of transient solubilizing modifications that are also employed in the synthesis of membrane peptides and proteins.^[117]^ In this context, the use of alternative ligation strategies such as the STL, DSL, and direct aminolysis methods have become common, as palmitoyl thioesters are often unstable in the presence of thiol additives used to enhance the rates of NCL reactions.[^118^, ^119^, ^120^]

**Scheme 2:**
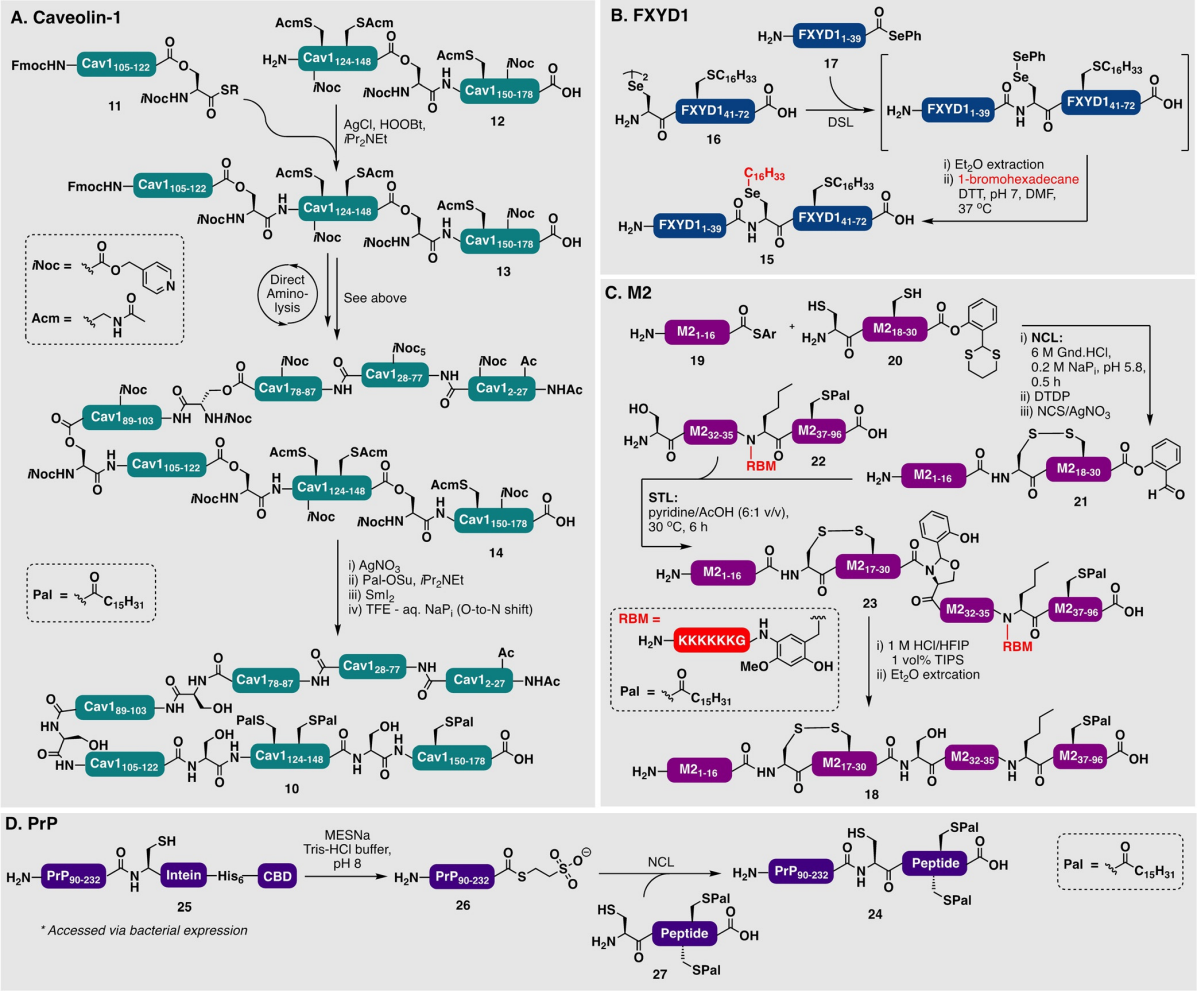
A) Direct aminolysis‐based assembly of palmitoylated caveolin‐1 (**10**) by Hirabayashi and co‐workers.^[120]^ B) Synthesis of a lipidated FXYD1 (**15**) through tandem DSL‐late‐stage alkylation by Chisholm et al.^[89]^ C) Synthesis of the palmitoylated M2 ion channel (**18**) by Huang et al. by using an RBM‐assisted STL approach.^[118]^ D) Semisynthesis of palmitoylated PrP (**24**) by Becker and co‐workers.[^55^, ^123^]

Numerous palmitoylated proteins have been accessed by total chemical synthesis to date. For example, Palà‐Pujadas et al. used an impressive five‐segment kinetically controlled NCL strategy to access the 175‐residue palmitoylated N‐terminal domain of the human Sonic Hedgehog protein.^[115]^ Hirabayashi and co‐workers performed a total synthesis and structural characterization of the 178‐residue caveolin‐1 (**10**), which is triply S‐palmitoylated in the C‐terminal region at Cys_133_, Cys_143_, and Cys_156_ (Scheme 2 A).^[120]^ Retrosynthetically, the protein was divided into five peptide segments which were fused using four consecutive ligation reactions. The synthesis proceeded through initial direct aminolysis between isopeptide fragments **11** and **12** to generate intermediate **13**. This was followed by iterative ligation reactions, affording the fully protected caveolin‐1 primary sequence (**14**). Finally, chemoselective deprotection of Acm, palmitoylation of the three deprotected Cys residues [using the electrophilic *N*‐succinimidyl palmitate (Pal‐OSu) reagent] and global deprotection furnished the target lipidated protein **10** (Scheme 2 A). It is important to note that the group observed that the peptide segments derived from caveolin‐1 were highly insoluble in aqueous buffer. Solubilizing *O*‐acyl isopeptide linkages were, therefore, employed to improve the solubility of the segments that were converted back into native peptide bonds following protein assembly. Furthermore, to bypass the need for aqueous solvents, the group utilized direct aminolysis reactions (rather than more traditional ligation approaches) to condense each fragment using DMSO as a solvent. These conditions are favorable for the stability of the palmitoyl thioesters, which are otherwise labile in aqueous media over long periods of time. However, direct aminolysis is not typically the method of choice of the practitioner due to the potential for 1) regioselectivity issues when other nucleophilic residues are present on the peptide fragment (e.g. Lys and Cys residues) and 2) epimerization of the α‐center at the ligation junction upon activation of the C‐terminal residue of one of the fragments. Regioselectivity issues can be avoided by using suitable protecting groups on the Lys and Cys residues, and epimerization can be avoided by judicious choice of ligation sites bearing only Gly, Pro, or isopeptide‐derived Ser residues on their N‐terminal side.^[120]^

In another example, Chisholm et al. accessed the membrane protein phospholemman (FXYD1)_1–72_ (**15**) using “reductive DSL” chemistry at low concentration to ensure solubility of a lipopeptide fragment **16** bearing a palmitylated Cys_42_ residue (Scheme 2 B).^[89]^ After the DSL reaction between **16** and a FXYD1_1–39_ N‐terminal selenoester **17**, the Sec residue at the ligation junction (Sec_40_) was subjected to late‐stage alkylation with 1‐bromohexadecane to afford a dipalmitylated analogue of the protein.^[89]^ Hanna et al. accessed di‐ and tri‐palmitoylated variants of the *Mycobacterium tuberculosis*‐associated antigen protein ESAT6 using a four‐component DSL/NCL strategy.^[121]^ Here, the N‐terminal ligation fragment was palmitoylated through direct coupling of the lipidated amino acid and the fragment was later converted into a C‐terminal thioester through a side‐chain anchoring strategy.^[122]^ A combination of DSL and NCL chemistry was used to prepare the majority of the ESAT6_17–95_ protein, which was then fused to the N‐terminal palmitoylated sequences in a final NCL reaction.

In a final key demonstration of these synthetic approaches, Huang et al. utilized a removeable‐backbone‐modification (RBM) tag to introduce solubility to a lipopeptide fragment for the synthesis of rabbit S‐palmitoylated sarcolipin (SLN) and S‐palmitoylated influenza A virus matrix‐2 (M2)_1–96_ (**18**) ion channel proteins using STL chemistry. The RBM used in this study, a 2‐hydroxy‐3‐methoxy‐4‐amidobenzyl group, bearing a 4‐amidohexalysine moiety to engender solubility, highlights the utility of solubility tags during the synthesis of palmitoylated fragments.^[119]^ The STL method is particularly attractive for the synthesis of these targets given the reaction does not require a thiol additive (unlike NCL), which would otherwise thiolyze the palmitoyl thioesters. To further expand on this work, Huang et al. developed a method to enable sequential NCL‐STL reactions in the N‐to‐C direction, which was subsequently employed to assemble the same S‐palmitoylated M2 ion channel target (**18**; Scheme 2 C), together with an S‐palmitoylated interferon‐induced transmembrane protein 3 (S‐palm IFITM3).^[118]^ The synthesis of **18** was achieved through an initial NCL reaction between thioester **19** and a cysteinyl fragment **20** (bearing a masked SAL ester). C‐Terminal activation through treatment with *N*‐chlorosuccinimide (NCS)/AgNO_3_ generated the SAL active ester intermediate **21**, which was reacted with the hydrophobic serinyl fragment **22** bearing the RBM (to aid solubility) under STL conditions to afford the M2_1–96_ precursor **23**. Target M2 protein **18** was generated through a final acidolysis of the remnant SAL oxazolidine.

Semisynthetic approaches provide a powerful means to access larger lipid‐modified proteins and, importantly, can overcome the need for multiple ligation steps that are usually necessary when accessing proteins of more than 100 residues in length by total chemical synthesis. Although semisynthetic approaches have been used widely for the generation of modified proteins, there are limited examples with palmitoylated proteins. One notable example, however, was the generation of palmitoylated variants of the mouse prion protein (PrP; **24**) using an EPL strategy (Scheme 2 D).[^55^, ^123^] The semisynthetic strategy relied on expression of recombinant murine PrP_(90–232)_ fragment **25** in fusion with a Mxe GyrA mini‐intein and two affinity tags (His_6_‐tag and chitin‐binding domain).

Mercaptoethanesulfonate sodium salt (MESNa) was used to intercept the scissile Ser−Cys amide bond between the intein and the remainder of the rPrP protein, thereby generating the corresponding MESNa thioester **26**. This was then used in a thiophenol‐promoted ligation reaction with the palmitoylated peptide membrane anchor fragment **27** to provide the target lipidated protein **24**. By using this strategy, five different palmitoylated variants were prepared in about 30 % yield and used to study the impact of lipidated PrP on the membrane structure and protein distribution in the membrane.^[124]^ This approach was later elaborated for the construction of PrP variants with either N‐terminal or centrally located truncations.^[123]^

## 4. Synthesis of Myristoylated Peptides and Proteins

**Scheme 3:**
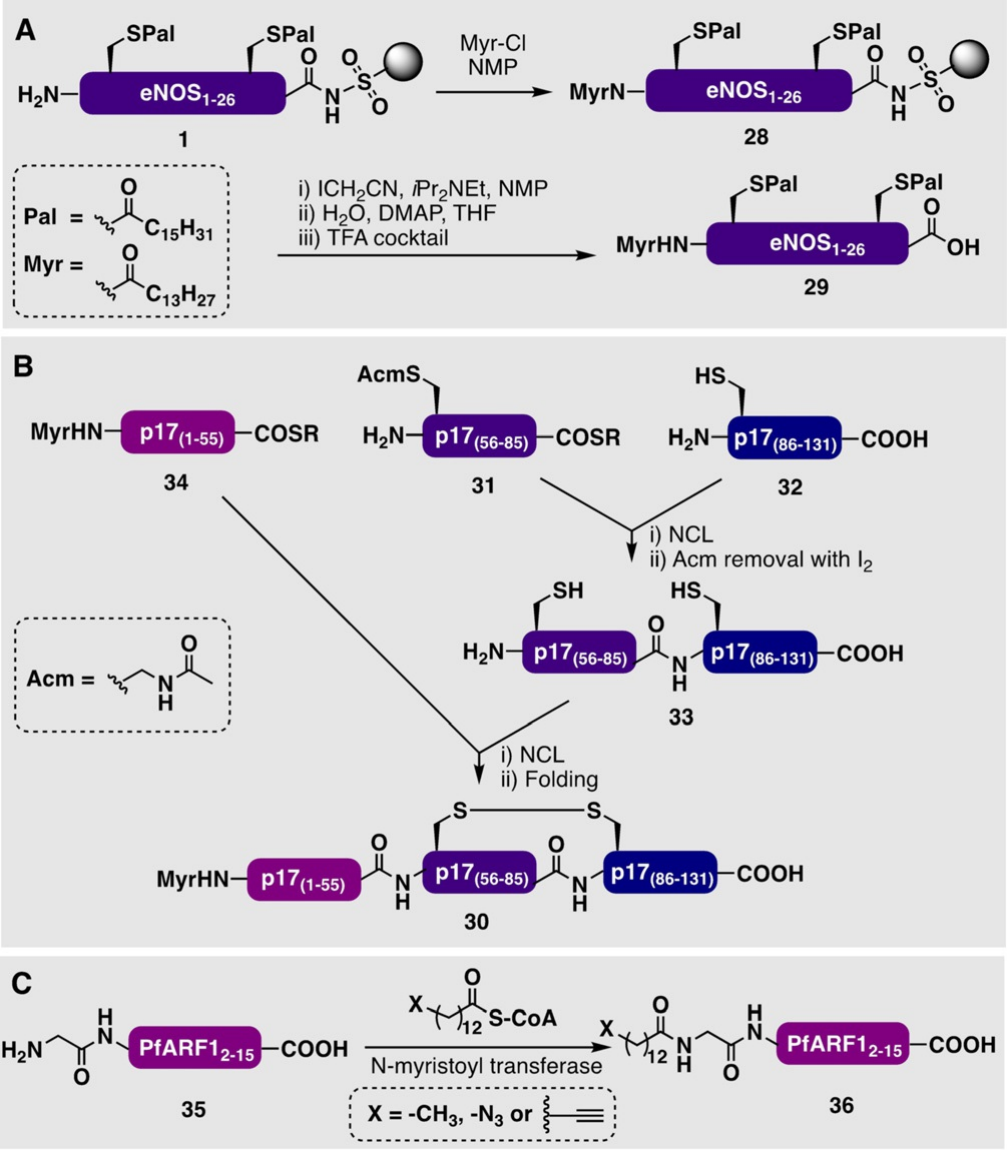
A) Synthesis of myristoylated and palmitoylated eNOS_1–26_ (**29**) by a solid‐phase approach developed by Waldmann and co‐workers.^[113]^ B) Synthesis of myristoyl‐p17_1–131_ (**30**) by a three fragment NCL approach by Lu and co‐workers.^[129]^ C) Metabolic incorporation of *N*‐myristoyl analogues into PfARF1_1–15_ (**36**) by Tate and co‐workers.^[130]^

N‐terminal myristoylation is typically achieved by the direct coupling of a fatty acid or pre‐activated fatty ester to the N‐terminal amine of a protected peptide during SPPS. Given their relative ease of synthesis, there is an abundance of examples in the literature of short, myristoylated peptides that have been produced synthetically.[^125^, ^126^, ^127^] For example, Waldmann and co‐workers prepared eNOS_1–26_, N‐myristoylated and S‐palmitoylated at two positions using a combination of orthogonal enzyme‐labile, acid‐labile, and noble‐metal‐labile protecting groups in a fragment condensation approach.^[128]^ However, this approach was low yielding (<1 %) and laborious, which led the group to develop a linear solid‐phase approach that made use of the Ellman‐sulfonamide resin linker (Scheme 3 A).^[113]^ Specifically, the authors expanded upon their solid‐phase synthesis of resin‐bound palmitoylated eNOS_1–16_ (**1**; Scheme 1 A), with a final Fmoc‐deprotection and on‐resin myristoylation with myristoyl chloride (Myr‐Cl) to assemble the full‐length resin‐bound precursor **28**. After alkylation of the resin linker (with iodoacetonitrile) and subsequent cleavage, this strategy provided the target myristoylated and dipalmitoylated eNOS_1–26_ peptide **29** in a much‐improved yield of 24 % after purification. Importantly, 25 mg of the eNOS peptide **29** could be prepared by this strategy and could be achieved in days to weeks, rather than months.

Access to larger myristoylated proteins is typically achieved by either semisynthesis or multicomponent ligation strategies. In an example of a protein prepared by chemical synthesis, Lu and co‐workers accessed the 131‐residue N‐myristoylated HIV‐1 matrix protein p17_1–131_ (**30**) by a three‐segment convergent NCL strategy (Scheme 3 B). To access each peptide fragment, the group utilized the in situ neutralization method developed by Kent and co‐workers for SPPS based on the Boc strategy.^[129]^ Initially, the internal and Acm‐protected thioester fragment **31** was ligated to C‐terminal cysteinyl fragment **32** to afford the non‐lipidated C‐terminus of the protein (**33**). A final NCL reaction with the myristoylated N‐terminal fragment **34**, derivatized as a thioester, then provided the final myristoyl‐p17 protein (**30**). Importantly, access to this synthetic myristoylated HIV‐1 matrix protein **30** allowed the authors to study the “myristoyl switch” hypothesis, which relates to the ability of the protein to interact with the cell membrane in a reversible manner.

Chemoenzymatic and metabolic approaches have also been used to access *N*‐myristoyl analogues substituted with terminal azide or alkyne functionalities, as exemplified by the enzymatic myristoylation of PfARF1_1–15_ (**35**) by Tate and co‐workers to produce both myristoylated and azido‐myristoyl derivatives of the PfARF1_1–15_ (**36**; Scheme 3 C).[^130^, ^131^, ^132^] These reactions are catalyzed by *N*‐myristoyltransferases which recognize the N‐terminal GXXXS motif and, therefore, allow the fluorescent labeling and imaging of myristoylated proteins within cells. These approaches have been reviewed previously and will not be discussed any further here.^[133]^

## 5. Synthesis of Prenylated Peptides

Like other lipidated peptides, the most common method for accessing prenylated peptides is by Fmoc SPPS‐based procedures. The lipid can either be incorporated directly on‐resin through the use of a pre‐prenylated amino acid building block^[134]^ or, alternatively, peptide precursors can be lipidated after cleavage of the resin through solution‐phase alkylation reactions.[^135^, ^136^, ^137^, ^138^] The reactive nature of the prenyl modification and its tendency to isomerize can present a number of challenges during synthesis. For example, the alkene functionality is easily degraded under acidic or reducing conditions, hence acid‐labile or hydrogenolytically labile resin linkers or protecting groups are not suitable for use with prenylated peptides.^[116]^ Moreover, prenyl groups must be compatible with the coupling conditions of the lipidated Cys building block.^[116]^ As Cys is prone to racemization, coupling conditions have been extensively studied and optimized. Importantly, it has been shown that a 1:1 mixture of HBTU/HOBt or HCTU with trimethylpyridine (TMP) as a base in CH_2_Cl_2_/DMF (1:1 v/v) leads to minimal racemization of the residue when coupling to the solid phase.[^64^, ^139^, ^140^]

**Scheme 4:**
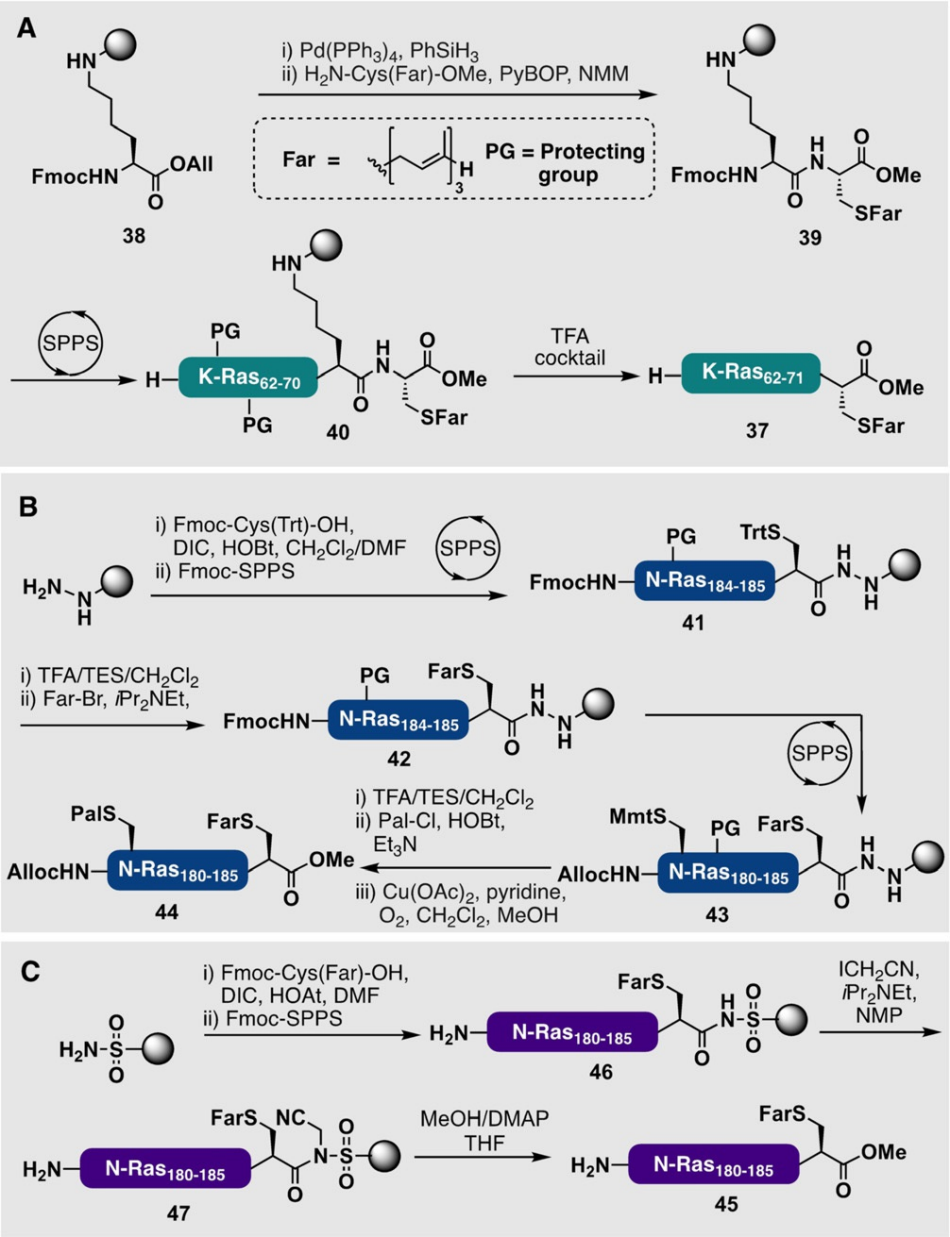
A) Synthesis of farnesylated K‐Ras 4B peptide (**37**) methyl ester through a side chain anchoring strategy by direct coupling of the C‐terminal lipidated residue.^[142]^ B) Use of an acyl hydrazide resin linker to access differentially palmitoylated and farnesylated N‐Ras_180–186_ methyl ester (**44**) on an oxidatively labile resin linker.^[136]^ C) Use of the Ellman sulfonamide linker to access farnesylated N‐Ras_180–186_ methyl ester (**45**) after alkylation and cleavage.^[113]^

A common strategy for the synthesis of prenylated peptides relies on the use of hyper‐acid‐labile 2‐chlorotrityl chloride (2‐CTC) resin linkers. This approach enables cleavage of the lipopeptide from resin using very mildly acidic conditions [for example, 1 vol % TFA or fluorinated alcohols such as trifluoroethanol (TFE) or hexafluoroisopropanol (HFIP)], which are compatible with the prenyl modifications. The only drawback of this strategy is that cleavage from the 2‐CTC resin liberates a C‐terminal carboxylic acid, whereas most prenylated proteins natively possess a C‐terminal methyl ester. Conveniently, peptides can instead be anchored to the resin through the side chain of an amino acid bearing a functionalizable side chain, which allows an appropriately amino acid methyl ester to be coupled to the C‐terminus. By using this approach, Waldmann and co‐workers prepared a farnesylated K‐Ras peptide 4B methyl ester (**37**) starting from side‐chain anchored Fmoc‐Lys‐OAll (**38**; Scheme 4 A).[^141^, ^142^] From here, deprotection of the allyl ester and subsequent coupling with H_2_N‐Cys(Far)‐OMe generated resin‐bound lipopeptide **39**. This farnesylated dipeptide was subsequently elongated by Fmoc‐SPPS to construct the full length farnesylated K‐Ras 4B (**40**) on the resin, before a final acidolytic cleavage afforded the target peptide **37** in 11 % overall yield.

An alternative approach to generating the C‐terminal methyl ester is to employ an oxidatively labile hydrazide linker which enables cleavage of the peptide under oxidative conditions that are orthogonal to standard Fmoc‐SPPS protecting groups. In this manner, Waldmann and co‐workers demonstrated the first differential lipidation of a peptide on an acid‐stable solid‐phase by selective deprotection of a Cys(Trt)‐containing intermediate **41** under mild acidolytic conditions and a subsequent alkylation with farnesyl bromide to provide **42** (Scheme 4 B).^[136]^ Extension by Fmoc‐SPPS including the coupling of Cys with orthogonal protection of the side chain by monomethoxytrityl (Mmt) provided resin‐bound intermediate **43**, which was selectively deprotected and acylated with palmitoyl chloride. A final oxidative cleavage from the resin in the presence of methanol provided the palmitoylated and farnesylated N‐Ras_180–186_ (**44**) as the corresponding C‐terminal methyl ester. The Ellman sulfonamide linker has also found utility for the preparation of lipopeptide thioesters bearing prenyl and fatty acyl groups. The benefit of this linker for use in this scenario is that it is very stable to both acid and base treatment, but the sulfonamide linker can be selectively alkylated, for example, with iodoacetonitrile, to activate the linker for cleavage. Specifically, alkylation of the sulfonamide renders the carbonyl moiety electrophilic and, as such, can be reacted with nucleophiles for modification of C‐terminal peptides. In the case of farnesylated N‐Ras_180–186_ (**45**), Waldmann and co‐workers demonstrated that after the generation of resin‐bound farnesyl‐peptide **46** and subsequent alkylation of the linker with iodoacetonitrile to provide **47**, methanol could be used to generate the target N‐Ras_180–186_ C‐terminal methyl ester **45** (Scheme 4 C).[^113^, ^116^] It should be noted, however, that undesired alkylation reactions elsewhere on the peptide can occur when alkylating the sulfonamide linker and, as a result, can lead to diminished yields. It has been shown that similar cassette strategies to those outlined above can also be adapted to the synthesis of geranylgeranylated peptides through alkylation of suitable peptide substrates with geranylgeranyl halides.^[143]^

**Scheme 5:**
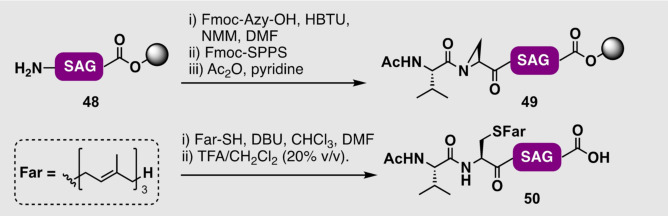
Aziridine‐mediated alkylation on‐resin by Gin and co‐workers.^[144]^

In an alternative approach it has been shown that aziridine‐2‐carboxylic acids can be incorporated into peptide sequences by SPPS, and subsequent site‐ and stereoselective opening can be performed on‐resin with suitable thiol nucleophiles such as farnesyl thiol to generate prenylated peptides. This elegant approach was showcased by Gin and co‐workers, whereby Fmoc‐aziridine‐2‐carboxylic acid (Fmoc‐Azy‐OH) was installed on a resin‐bound tripeptide **48** using Fmoc‐SPPS to afford pentapeptide **49** (Scheme 5).^[144]^ Interception of the aziridine functionality with farnesyl thiol under basic conditions, prior to cleavage of the resin and deprotection, then provided S‐farnesylated peptide **50**. This approach, however, has yet to be demonstrated on larger peptidic systems and the applicability in the presence of all proteinogenic amino acids (e.g. Cys residues that may cross‐react with the aziridine moiety) has not yet been explored.

## 6. Synthesis and Semisynthesis of Prenylated Proteins

One group of prenylated proteins that have been intensively studied belongs to the Ras superfamily of GTPases, with a number of important biological studies underpinned by the ability to access pure versions of these modified proteins through semisynthetic methods.^[116]^ The Ras superfamily belongs to the class of monomeric G proteins that are involved in many cellular processes, including signal transduction and cell‐cycle regulation.^[145]^ They can switch between an active, GTP‐bound state and an inactive, GDP‐bound state. In their active form, they can either stimulate or inhibit cellular processes through interaction with various effectors.^[64]^ As they are important molecular switches, dysregulation of Ras proteins is highly relevant in the development of cancer.^[146]^ For this reason, the genes that code for these proteins are some of the most important human oncogenes^[147]^ and continuously activated Ras proteins are found in 30 % of all solid human tumours.[^64^, ^148^] The best studied proteins in the family are the three isoforms K‐Ras, N‐Ras, and H‐Ras,^[116]^ which share approximately 90 % sequence identity in the first 168 residues and most of the variation between them arises in the C‐terminal region (20 residues), which also contains sites of post‐translational lipidation.^[146]^ The three most common types of lipidation found in Ras proteins are S‐palmitoylation, S‐prenylation, and N‐myristoylation. These lipid modifications lead to the association of Ras proteins to membranes and are essential for their function.

**Scheme 6:**
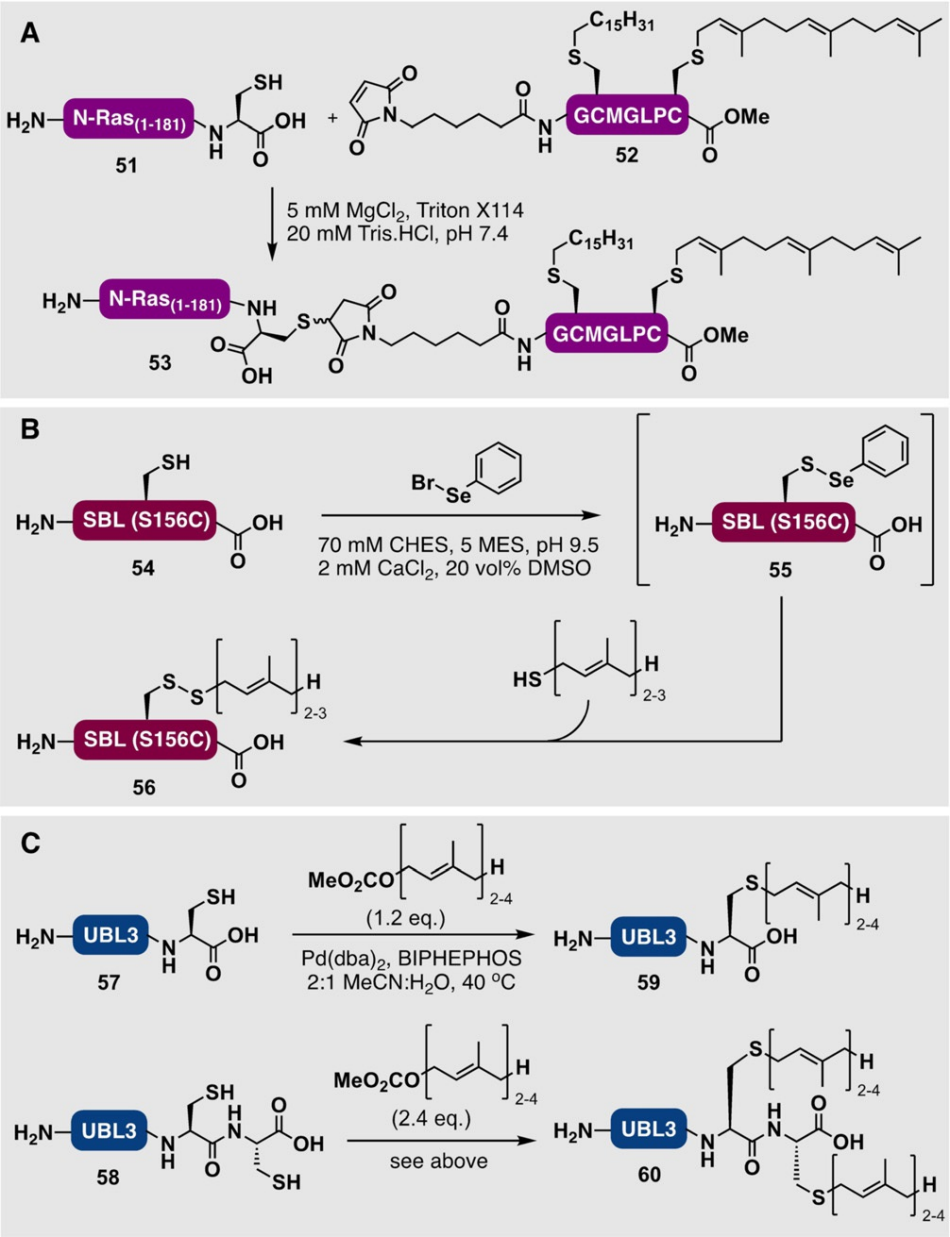
A) MIC‐based ligation assembly of a farnesylated N‐Ras analogue **53** by Waldmann and co‐workers.^[149]^ B) Late‐stage prenylation of a cysteinyl subtilisin *Bacillus lentus* (SBL) mutant **54** via intermediary protein selenylsulfide **55** by Davis and co‐workers.^[162]^ C) Pd‐catalyzed Tsuji–Trost allylation for the single or double prenylation of UBL3 [bearing one (**57**) or two (**58**) C‐terminal Cys residues] by Becker and co‐workers.^[163]^

Given the difficulty associated with isolating the full‐length lipidated Ras proteins, many early structural and biochemical studies were performed exclusively with the soluble domain missing the unstructured C‐terminal portion.^[116]^ To better understand the effect of prenylation on the activity of Ras proteins, a number of groups subsequently developed several powerful methods to access natively modified Ras proteins. In the early 2000s, efforts by the Kuhlmann and Waldmann groups focused on using an expressed protein MIC‐ligation between an expressed N‐Ras_1–181_ protein **51** (bearing a C‐terminal Cys) and a lipidated maleimidyl peptide **52** to access both palmitoylated and farnesylated N‐Ras_1–181_ (**53**) with a non‐native maleimide linker (Scheme 6 A).[^149^, ^150^] Irrespective of the non‐native linker, these mimics could be efficiently incorporated into artificial membranes and exhibited affinity for effector proteins in vivo.[^68^, ^151^] Furthermore, these semisynthetic proteins were used to study the palmitoylation cycle of N‐Ras in cells.^[26]^ The N‐Ras protein was also equipped with a photoactivatable geranylbenzophenone analogue of the farnesyl modification, which was subsequently used to interrogate protein–protein and protein–lipid interactions in cells.^[151]^ Following the landmark studies from the Kuhlmann, Bastiaens, and Waldmann groups using MIC ligations,[^68^, ^152^] the groups of Goody and Waldmann reported the first synthesis of a native geranylgeranylated Rab7 protein by an intein‐mediated EPL strategy.^[153]^ Briefly, they prepared a recombinant Rab7 protein segment C‐terminally fused to an intein, which was cleaved by incubation with MESNa to provide the corresponding thioester. This Rab7 thioester segment was ligated to a synthetic geranylgeranylated N‐cysteinyl peptide. This enormously powerful EPL strategy has since been used to access geranylgeranylated Ypt1 GTPase,^[154]^ prenylated Rab7,^[143]^ mono‐/digeranylgeranylated Rab7,^[155]^ farnesylated Rheb and K‐Ras4B,[^94^, ^156^] and farnesylated Rheb proteins.^[157]^ In most of these cases, finding a suitable detergent to enable the ligations to proceed at sufficient reaction rates and to keep the prenylated peptides and proteins solubilized in aqueous buffers was crucial.[^143^, ^158^] It should be noted that, to date, Rab proteins have primarily been the targets of the EPL approach to access C‐terminally lipidated proteins. This is in large part due to the availability of “solubilizing” binding partners for these proteins such as the Rab escorting protein REP‐1, which can be used to solubilize the resulting lipidated proteins during ligation and folding. Folding is the final and critical step in generating active, post‐translationally modified semisynthetic proteins. Two other solubilizing chaperone‐like proteins have also been applied to solubilize prenylated Rab proteins, namely, the GDP‐dissociation inhibitor (GDI)^[154]^ and the β‐subunit of RabGGTase to renature the geranylgeranylated protein Rab7.^[159]^ Indeed, although some Ras‐type proteins including K‐Ras4B and D‐Ral have been accessed using the EPL strategy,^[141]^ a chaperone is not widely available for many other members of the Ras protein family, which makes folding to the native form following ligation challenging.

Another avenue to access native prenylated proteins is through the late‐stage chemoselective modification of proteins. Despite progress in the field of site‐selective protein modification, there are still challenges to overcome with respect to the regioselectivity, chemoselectivity, and stability of the resulting proteins as well as the development of reactions that work efficiently in aqueous buffers at physiological pH and temperature to prevent denaturation of the target proteins.[^160^, ^161^] For this reason, there are fewer examples of prenylated proteins generated by late‐stage modification than for the ligation‐based strategies outlined earlier.^[160]^

An example of a late‐stage lipidation method was reported by Davis and co‐workers which exploited the unique reactivity of selenylsulfides for the thiol‐selective prenylation of proteins.^[162]^ Specifically, the authors were able to pre‐activate a S156C mutant of the model protein subtilisin *Bacillus lentus* (SBL; **54**) containing one exposed Cys as a phenyl selenylsulfide **55** through a reaction with phenylselenyl bromide (Scheme 6 B). This species was then reacted with a prenylated thiol in aqueous solution (containing 20 vol % DMSO to solubilize the highly hydrophobic prenyl thiols), which led to the formation of the asymmetric prenyl‐protein disulfide‐linked construct **56**. In a similar fashion, both farnesyl and geranyl modifications of subtilisin were obtained (with >50 % and >90 % conversion, respectively). It should be noted that it was not possible to install the geranylgeranylation modification by this approach, most likely because of the insolubility of the geranylgeranyl thiol in the aqueous buffer systems required to solubilize the protein. A potential drawback of this method is the non‐native disulfide linkage to the lipid modification, which can be cleaved under reducing conditions.^[162]^ Very recently, Becker, Breinbauer, and co‐workers reported a method for the late‐stage prenylation of expressed proteins by Pd‐catalyzed Tsuji–Trost allylation (Scheme 6 C). By using this approach, the authors were able to install farnesyl, geranyl, geranylgeranyl, and other non‐native cargos to the C‐terminal Cys residue of ubiquitin‐like protein 3 (UBL3). In this case protein variants bearing either one (**57**) or two (**58**) Cys residues were used, which afforded singly (**59**) or doubly (**60**) lipidated UBL3 proteins, respectively. Importantly, the prenyl modifications installed by the Tsuji–Trost allylation reaction possess a native thioether bond, which makes this approach a particularly promising new strategy for installing native prenyl modifications on peptides and proteins in solution.^[163]^

## 7. Synthesis of PE‐Linked Peptides and Proteins

**Scheme 7:**
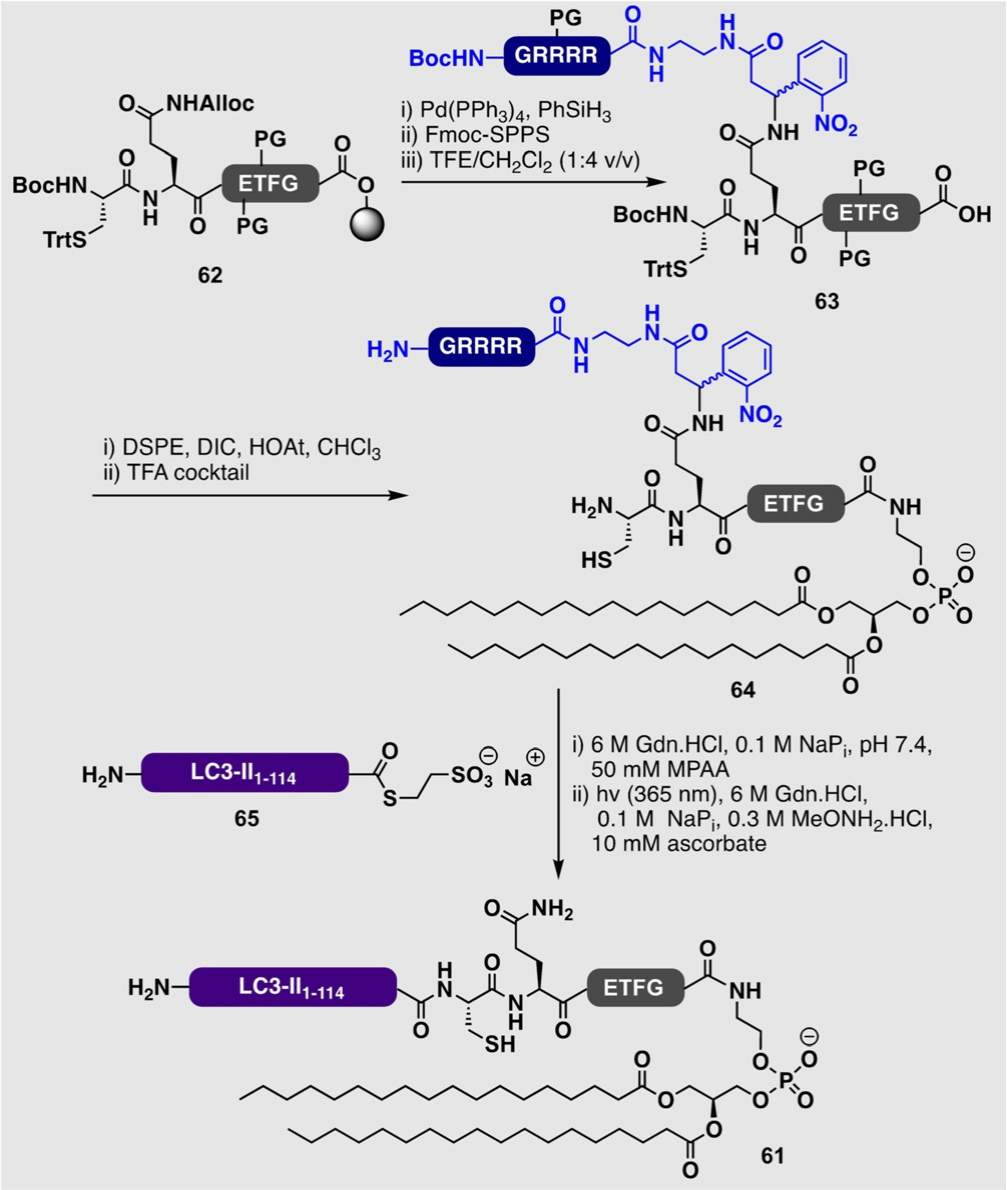
Synthesis of PE‐modified LC3‐II protein (**61**) by Huang et al., utilizing a photocleavable Arg‐rich solubility‐enhancing peptide on the Gln side chain (displayed in blue).^[164]^ DSPE=1,2‐distearoylphosphatidylethanolamine.

The synthetic addition of phosphatidylethanolamine (PE) to the C‐terminus of peptides and proteins has proven extremely challenging due to the very hydrophobic nature of the PE moiety. One notable example that addressed the solubility problem was reported by Liu and co‐workers, who accessed PE‐modified LC3‐II protein (**61**) in practical quantities.^[164]^ Key to their success was the implementation of a photolabile solubilizing tag installed on an orthogonally protected resin‐bound hexapeptide **62**. After cleavage from the resin under mild acidolytic conditions, the resulting intermediate **63** was then coupled to 1,2‐distearoylphosphatidylethanolamine (DSPE) in the presence of DIC/HOAt to afford **64**. A final NCL between this cysteinyl PE‐modified hexapeptide and an expressed LC3‐II MESNa thioester (**65**) (generated through intein thiolysis) provided access to the target PE‐linked protein **61** after UV‐mediated removal of the photolabile solubility tag (Scheme 7).

In the same year as the example above, Wu and co‐workers reported another semisynthesis of a PE‐modified LC3 protein by a similar EPL strategy, and the resulting lipid‐modified protein was used to study autophagy.^[165]^ In this case, the LC3 protein was expressed as an N‐terminal MBP and C‐terminal intein fusion construct in *E. coli*, and treatment with MESNa gave the MBP‐LC3 thioester with sufficient solubility to achieve the subsequent ligation to a PE‐carrying peptide. To access the native full‐length lipidated LC3, the MBP was eventually cleaved with TEV protease. The semisynthetic lipidated protein was shown to be functional through its interaction with the protease Atg4B and its activity in membrane tethering and fusion, which are key for the role of LC3 in autophagy.^[166]^

## 8. Synthesis of Cholesterol‐Linked Peptides and Proteins

The C‐terminal modification of proteins with a cholesterol molecule is responsible for controlling the localization of proteins at the cell membrane. One notable example is the hedgehog family proteins, which are commonly modified with a C‐terminal cholesterol moiety.^[167]^ Waldmann and co‐workers generated mimics of these cholesterol‐modified hedgehog proteins by using a MIC ligation strategy involving an expressed protein fragment and a smaller synthetic peptide fragment bearing a C‐terminal cholesterol moiety. Although these possess a non‐native maleimidyl linker, the constructs enabled the authors to perform key experiments that revealed the ability of cholesterol alone to anchor proteins to membranes with affinities comparable to dual lipidation motifs, such as S‐farnesylation with additional geranylgeranylation or S‐palmitoylation, found on other lipidated proteins.^[171]^

**Scheme 8:**
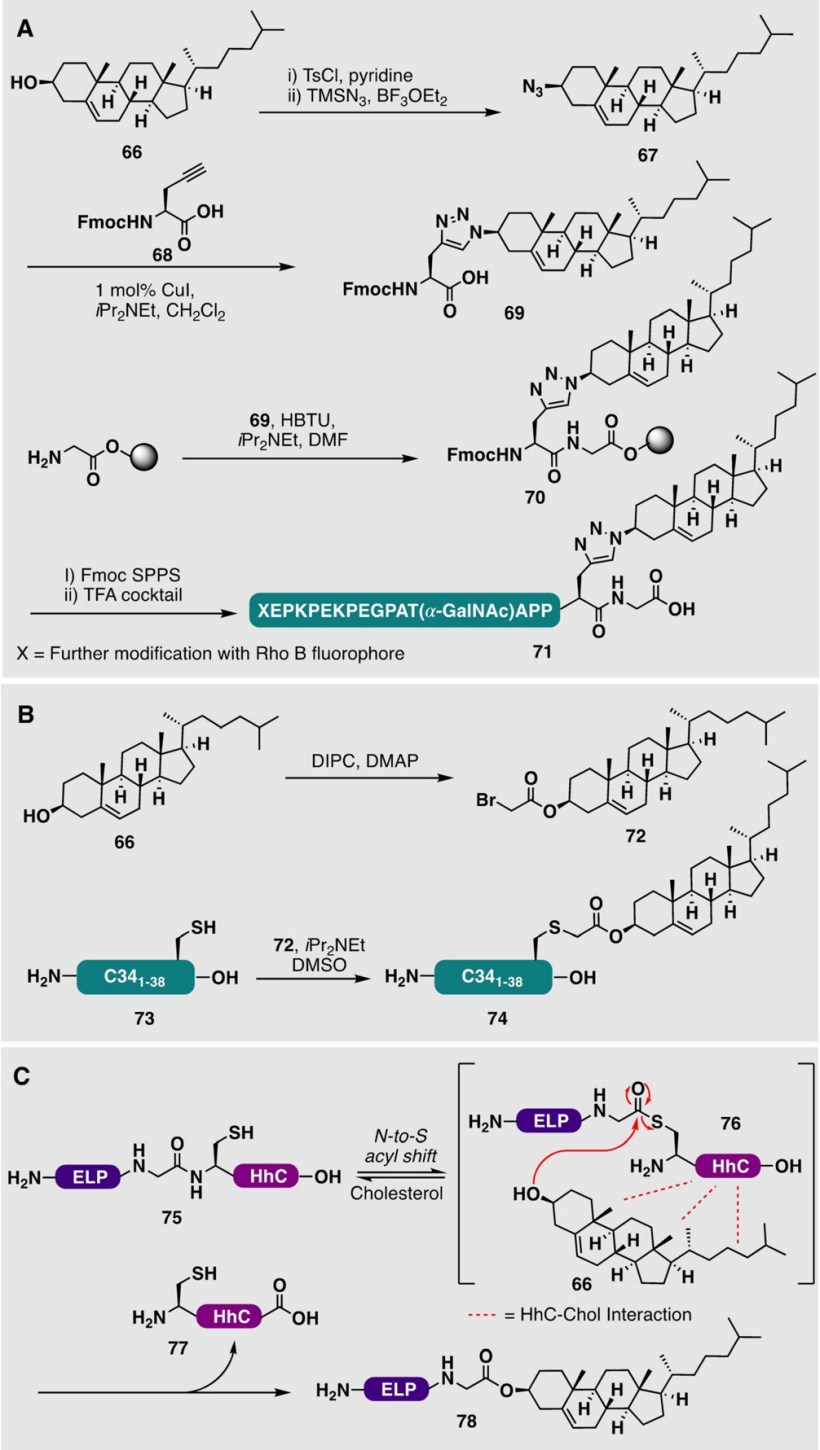
A) Synthesis and incorporation of an Fmoc‐protected triazole‐linked cholesteryl amino acid (**69**) by Blixt and co‐workers.^[168]^ X=further modification with the Rho B fluorophore. B) Derivatization of C34 peptide (**73**) with cholesterol by Ingallinella et al.^[169]^ C) Semisynthesis of a C‐terminally cholesterylated elastin‐like polypeptide (ELP) (**78**) by Chilkoti and co‐workers through fusion of ELP to a cholesterol‐binding HhC protein (**75**).^[170]^

Teruya et al. have also reported the semisynthesis of GFP bearing a C‐terminal cholesterol moiety as a model system. To access this, the group used a GFP‐thioester prepared using intein technology. This was ligated to a small peptide bearing a C‐terminal cholesterol moiety, using a detergent to aid the solubility of the lipopeptide fragment. Confocal fluorescence microscopy was then used to study the localization of the protein within membranes.^[172]^ In a building block approach starting from cholesterol (**66**), Blixt and co‐workers synthesized an azide‐containing cholesterol derivative **67** for reaction with an alkynyl‐amino acid **68** to generate a triazole‐linked modified amino acid **69** (Scheme 8 A). The applicability of this cholesterylated cassette **69** was then demonstrated through coupling of the building block to resin to generate **70**, which could be elongated through standard Fmoc‐SPPS to generate glycosylated model lipopeptide **71**.^[168]^ Ingallinella et al. took a solution‐phase approach to the derivatization of cholesterol (**66**) through the reaction of cholesteryl bromide (**72**) with a C34 peptide **73**, bearing an unprotected C‐terminally positioned cysteine residue, to generate cholesterylated C34 (**74**; Scheme 8 B). This allowed the authors to increase the antiviral potency of HIV‐1 peptide fusion inhibitors by targeting it to the cell compartment where fusion occurs.^[169]^

Recently, Chilkoti and co‐workers developed an elegant enzymatic method for accessing C‐terminal cholesterol‐modified peptides and proteins, such as elastin‐like polypeptide (ELP).^[173]^ This strategy involved the fusion of ELP to a secondary HhC protein (autoprocessing C‐terminal domain of hedgehog protein; **75**), which recognizes and binds cholesterol. Upon the binding of cholesterol (**66**), an intein‐like N‐to‐S acyl shift, involving the HINT domain shared between the hedgehog protein and inteins, forms a reactive thioester intermediate **76**. This thioester subsequently reacts with the 3β‐hydroxy group on an associated and proximal cholesterol (**66**) molecule, which ultimately results in the extrusion of the HhC domain (**77**) and formation of the cholesterol‐modified ELP (**78**; Scheme 8 C).^[170]^ The authors used this approach to attach cholesterol to the bioactive peptide exendin‐4, an approved peptide drug for type II diabetes. Importantly, the authors showed that the cholesterol modification led to self‐assembly of the peptide into micelles, which then activated the glucagon‐like peptide I receptor with high potency. Given the ability of cholesterol to direct biomolecules to specific sites on membranes, including ordered domains (rafts), it is anticipated that the methods described above will continue to find widespread use in a number of fields, ranging from chemical biology to drug discovery and delivery.[^174^, ^175^, ^176^]

## 9. Synthesis of GPI‐Linked Peptides and Proteins

The first total synthesis of the native GPI anchor molecule was reported in the late 1990s.^[177]^ Since this seminal report, several synthetic routes to the GPI anchor have been reported and have been reviewed elsewhere.[^178^, ^179^] Although the early syntheses of the GPI anchor represented substantial feats in synthetic organic chemistry, the molecules were not equipped with appropriate functionality for fusion to a peptide or protein. In a major advance in the field of lipidated protein synthesis, in 2004, Guo and co‐workers employed a convergent strategy to assemble a 12‐residue GPI‐anchored CD52 antigen peptide.^[180]^ The group accessed the CD52 glycopeptide and GPI anchor separately, then fused both fragments using an HOBt/EDC‐mediated coupling. This work was followed rapidly by reports detailing the synthesis of analogues of GPI‐anchored proteins, including a GFP‐GPI mimic^[181]^ and an EYFP‐GPI mimic.^[182]^ By using an alternative strategy, Guo and co‐workers employed a sortase A mediated ligation to modify peptides and small proteins with GPI anchors.^[183]^ However, this enzymatic approach suffered from two major drawbacks: 1) the attachment of one or two non‐native Gly residues to the phosphoethanolamine moiety of the GPI anchor was necessary for the recognition of sortase A, and 2) the recognition sequence (LPXTG) introduced into the protein C‐terminus resulted in a non‐removable and non‐native ligation scar in the final modified protein product. Nevertheless, this strategy could be employed for the efficient preparation of analogues of human CD52 and CD24 antigens as well as a GPI‐anchored MUC1, containing a short peptidic sequence of the tumor‐associated protein.

**Scheme 9:**
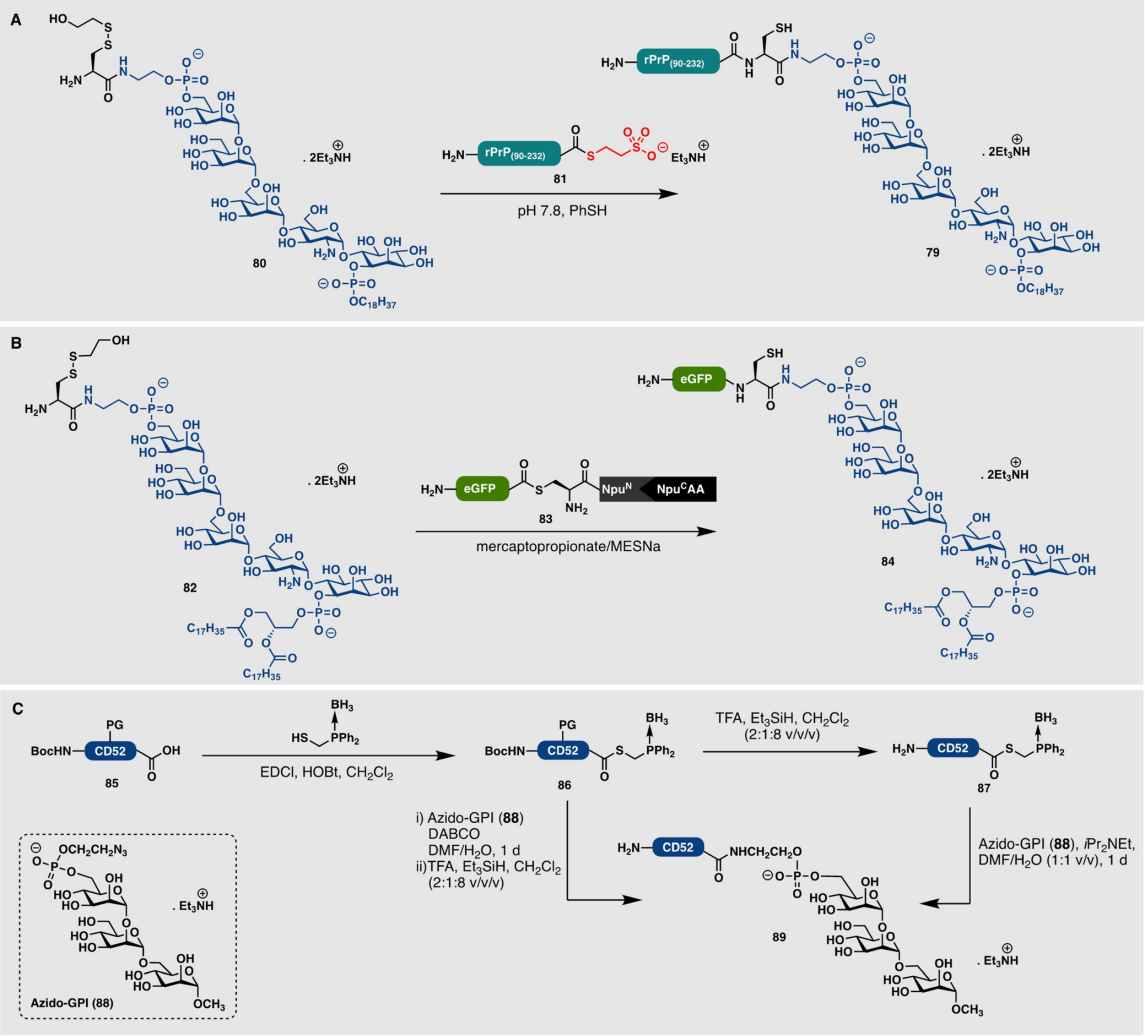
A) NCL‐based assembly of a Cys‐functionalized GPI anchor (blue) and a recombinant prion protein [rPrP_(90–232)_ bearing a C‐terminal MESNa thioester (red)] by Becker, Seeberger, and co‐workers.^[185]^ B) One‐pot NCL of a Cys‐functionalized GPI anchor (blue) with an eGFP‐Npu intein intermediate.^[186]^ C) Staudinger‐based synthesis of trimannose GPI‐modified CD52 by Guo and co‐workers.^[187]^ The peptide was synthesized by Fmoc‐SPPS on an acid‐sensitive 2‐CTC resin. Side chain protected CD52 was converted into its cognate phosphinothioester prior to Staudinger ligation with an azide‐functionalized GPI. PG=protecting group.

Another powerful approach for linking GPI anchors to peptides and proteins is through NCL. Nakahara and co‐workers were the first to link thioester peptides and Cys‐containing GPI using an NCL‐based approach,^[184]^ whereas Bertozzi and co‐workers used EPL to fuse GPI analogues to recombinant proteins, specifically using this strategy to access GPI‐modified GFP constructs that allowed them to probe the effect of these lipids on protein–membrane targeting and membrane diffusion.^[46]^ Building on these seminal studies, Becker, Seeberger, and co‐workers were able to develop a robust and generalizable semisynthetic strategy for the preparation of homogeneously GPI‐anchored recombinant prion protein (rPrP; **79**) based on an NCL platform.^[185]^ Specifically, a synthetic Cys‐tagged GPI anchor (**80**) was ligated to an expressed rPrP bearing a C‐terminal MESNa thioester (**81**) by NCL (Scheme 9 A). The ligation was performed at pH 7.8 in the presence of a thiophenol as a thiol additive, which led to the efficient generation of the GPI‐anchored protein. Notably, no addition of detergents or lipids was required during the ligation (which was performed in standard 6 M Gdn⋅HCl, 0.3 M NaP_i_ buffer) and the excess GPI anchor could be recovered and recycled after the reaction. Recently, Varón Silva and co‐workers further improved on this approach by the integration of a one‐pot ligation strategy to semisynthetically access complex GPI‐anchored proteins (Scheme 9 B).^[186]^ For example, a similar synthetic Cys‐containing GPI anchor (**82**) could be ligated with an active eGFP protein thioester formed in situ from the respective protein‐Npu intein intermediate (**83**) to generate homogeneous GPI‐anchored eGFP (**84**). A similar strategy has also been used for the successful semisynthesis of Thy1 and *Plasmodium berghei ANKA* MSP119 proteins, both of which bear homogeneous GPI anchors, although extended reaction times were necessary.

Although both sortase A and NCL‐based ligation strategies provide an efficient means to link synthetic GPI molecules to proteins, each requires the use of non‐native protein–GPI linkages, through either an additional peptidic recognition sequence (in the case of sortase ligation) or a remnant Cys residue following the NCL step. With this in mind, and a view to generating truly native protein–GPI constructs, Zhu and Guo developed a method for coupling GPI to peptides and proteins through the use of the traceless Staudinger ligation (Scheme 9 C).^[187]^

In an example of this approach, the CD52 peptide (**85**) was synthesized by Fmoc‐SPPS on a hyper‐acid‐sensitive 2‐CTC resin and, following cleavage from the resin, was converted into the respective phosphinothioester (**86**); this could be further deprotected upon acid treatment to afford phosphinothioester **87**. Ligation of either **86** or **87** with azide‐functionalized GPI (**88**) proceeded smoothly, thereby providing the human CD52 antigen bearing a fully native linkage between the protein and the GPI anchors (**89**). The strategic use of the traceless Staudinger ligation in this manner sets the scene for the generation of many more native GPI‐anchored proteins in the future, including important proteins such as the CD48 antigen and carbonic anhydrase IV (both GPI‐anchored through a C‐terminal Ser) or the Eph receptor ligand ephrin A5 (which is GPI‐anchored through a C‐terminal Asn), all of which have not been studied in homogeneous form to date. Key to such studies will be the implementation of recently developed predictive tools to identify new GPI‐anchored proteins, such as PredGPI,^[188]^ and the extension of phosphinothioester generation to larger and recombinant proteins, for which intein‐based methods could potentially prove an enabling technology. However, given that the traceless Staudinger ligation can suffer from slower reaction rates compared to other ligation methods, it is possible that such larger protein phosphinothioesters may not ligate as efficiently as the smaller peptide examples explored to date.^[189]^

A limiting factor in the approaches described above is the availability of sufficient amounts of functionalized GPI anchor, as these species are difficult to prepare by multistep synthetic routes. Furthermore, difficulties encountered while handling these native lipid‐modified proteins as a consequence of solubility problems and/or amphipathic properties has meant that many researchers have turned to the use of less‐complex GPI core structures in synthetic and semisynthetic campaigns.^[190]^ A potential solution is to harness natural GPI anchors made by cells; however, only a few examples of this approach for the generation of GPI‐anchored peptides have been reported to date. For example, Schumacher et al. described the generation of a GPI‐anchored peptide with a free N‐terminal Cys in yeast that can be used in ligation reactions with peptide or protein thioesters.^[191]^ Alternatively, Dhar and Mootz reported the innovative use of a split intein‐based (*Npu* DnaE) system that relies on expressed GPI‐anchored peptides fused to a C‐terminal intein segment, with subsequent trans‐splicing with another protein bearing an N‐terminal intein (in this case the model protein eGFP).^[192]^

## 10. Summary and Outlook

The methods and examples described in this Review summarize the current status of (semi‐)synthetic strategies to generate lipidated peptides and proteins. To provide an easily accessible picture of this field, we set out to provide a concise overview of the major classes of lipidation found on peptides and proteins. A succinct description of the most relevant chemical approaches to generate lipidated peptides and proteins has been provided. We highlight advantages and challenges of the individual strategies, which we further elaborate on when highlighting specific examples for each class of lipidation. From these examples, it is clear that our ability to assemble homogeneous lipid‐modified proteins from segments made by SPPS and recombinant expression has significantly matured over the past decade and has served as the basis for a number of important fundamental discoveries in biology and medicine.

However, the sensitive nature of linkages between lipids and proteins (e.g. thioesters), the chemical complexity of specific lipid modifications (e.g. GPI or PE anchors), and the impact of lipid modifications on ligation yields as a result of increased hydrophobicity or amphipathicity still make these synthetic and semisynthetic endeavors incredibly challenging. Although in some cases these limitations can be side‐stepped by introducing non‐native linkages between lipid(s) and proteins, this raises concerns about the functional consequences of introducing these artificial variations.

Despite significant progress over the past decade, as highlighted in this Review, our knowledge on the functional roles of different lipidation modifications, and patterns thereof, remains incomplete. However, it is envisaged that further extensions and improvements to ligation‐based protein synthesis methods such as NCL (and EPL), DSL, STL, and KAHA will continue to drive the field forward. One key requirement will be to perform ligation reactions at lower concentrations, which can be achieved with further extensions to the DSL method and by employing solubilization tags on lipidated peptide segments to improve NCL, EPL, and even protein trans‐splicing (PTS) reactions that rely on split inteins.[^2^, ^77^]

We anticipate that additional progress in the field will be made through the combination of the sophisticated ligation strategies described above with the development of novel chemo‐ and regioselective modification reactions, for example, lipidation of unprotected cysteine residues.[^193^, ^194^] There have been a number of new approaches recently developed towards this end, driven by the need for efficient conjugation reactions to generate selectively modified protein therapeutics. These methods can now be repurposed for late‐stage lipidation, thus avoiding handling problems during protein synthesis. Similarly, enzyme‐mediated strategies offer opportunities for the synthesis of lipidated proteins at two distinct steps. First, for protein assembly through the use of enzyme‐mediated ligation strategies (e.g. using engineered proteases or specific peptide ligases),^[195]^ and second for late‐stage enzymatic lipidation.^[196]^ The use of these emergent approaches, either together or in combination, should enable more efficient and robust access to lipidated proteins, thus accelerating efforts to study the roles of lipidation in fundamental biological studies, as well as to provide high‐quality lipidated proteins for the biotechnology and pharmaceutical sectors.

## Conflict of interest

The authors declare no conflict of interest.

## Biographical Information

*Cameron C. Hanna graduated in Chemistry from the University of Otago, New Zealand, in 2015. He then completed his PhD at the University of Sydney, Australia, in 2020 with Prof. Richard Payne, before postdoctoral research in the group of Prof. Dame Margaret Brimble. He is currently a postdoctoral research associate in the group of Prof. Bradley Pentelute at MIT, where he focuses on the development of peptide drug discovery platforms based on phage display as well as new methods to site‐selectively modify proteins on live cells*.

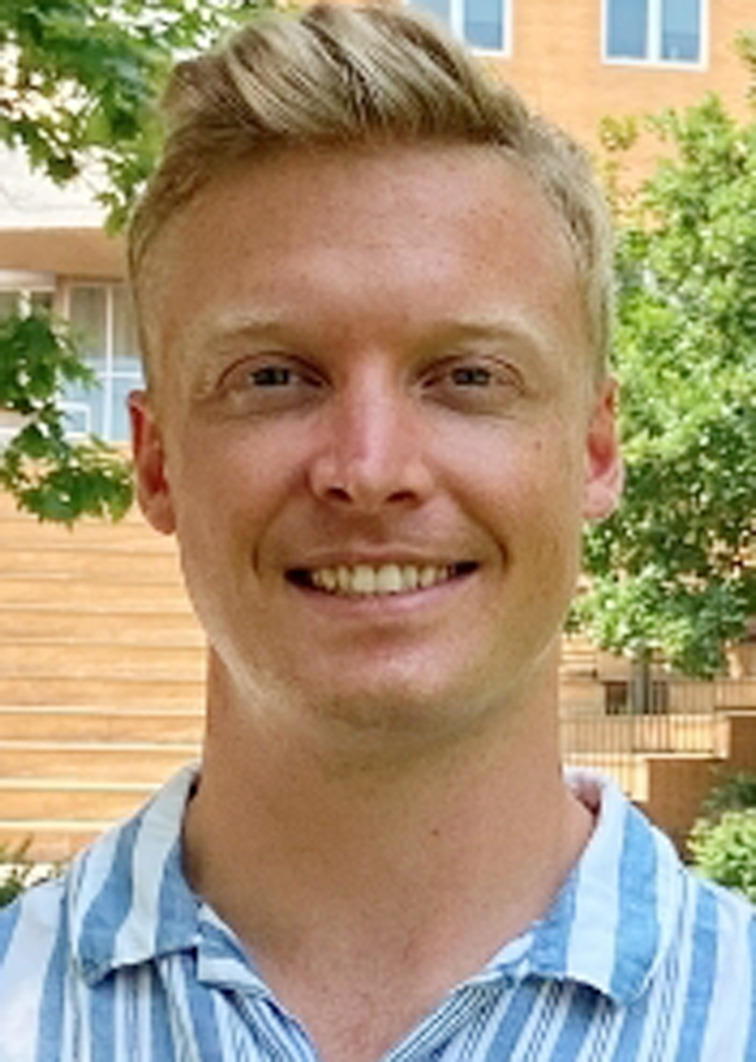

## Biographical Information

*Julia Kriegesmann received her MSc in Chemical Biology from the TU Dortmund, Germany in 2016 and her PhD from the University of Vienna, Austria, in 2021 under the supervision of Prof. Dr. Christian F. W. Becker. She is currently a postdoctoral researcher in the Becker research group, where she continues her work on the selective modification of cysteines within peptides and proteins*.

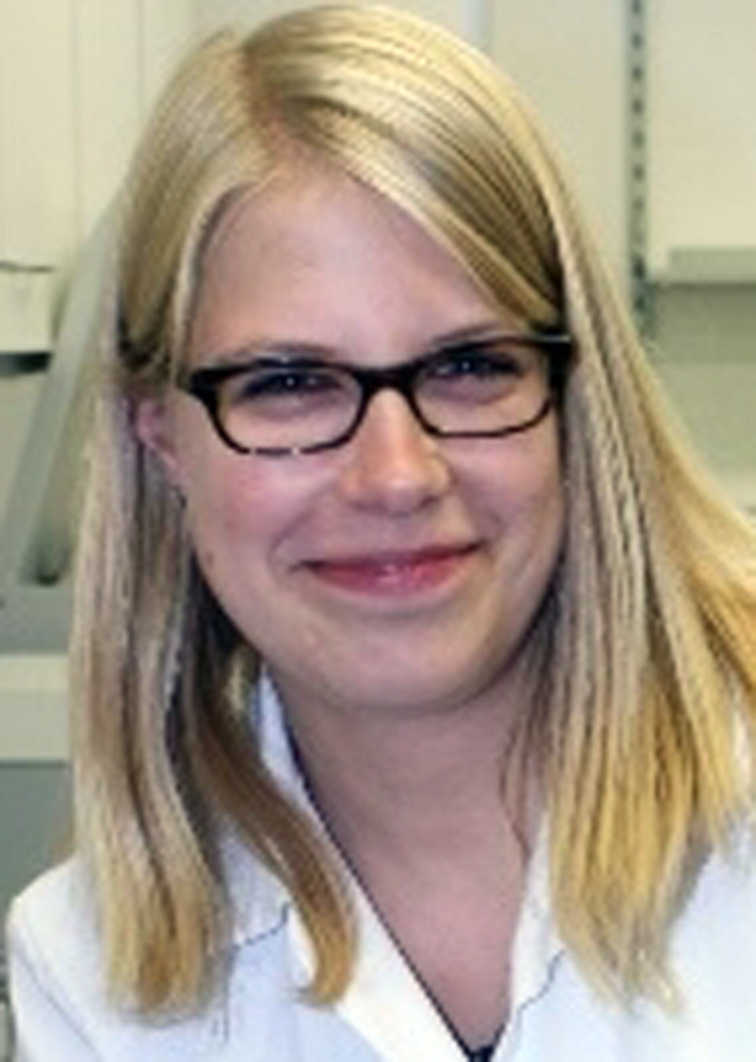

## Biographical Information

*Luke J. Dowman graduated in chemistry and biochemistry from The University of Sydney, Australia in 2015. He completed his PhD at The University of Sydney with Prof. Richard Payne in 2020. During this time, he focused on synthetic routes to access sulfopeptide analogues and the rapid and site‐selective functionalization of peptides and proteins. He is currently a postdoctoral research associate within the Payne group and the ARC Centre of Excellence for Innovations in Peptide and Protein Science, where he continues his research on new protein functionalization methods*.

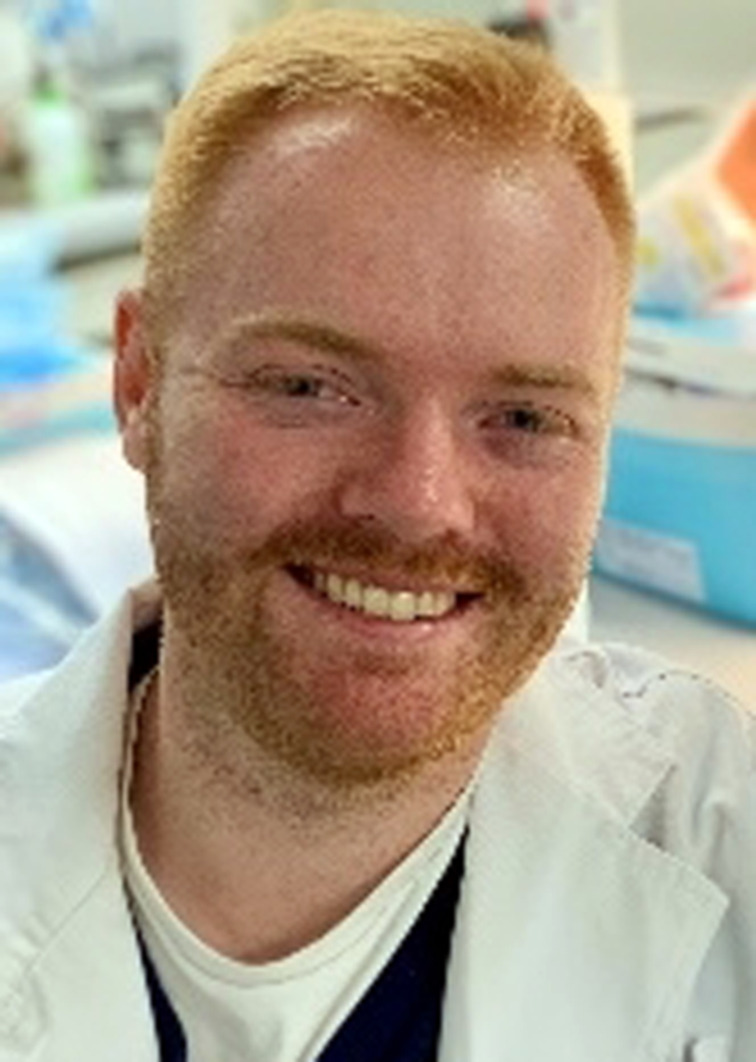

## Biographical Information

*Christian F. W. Becker studied chemistry (diploma 1998) at the University of Dortmund (Germany). After completing his PhD in 2001 at the same university, he carried out postdoctoral research at Gryphon Therapeutics (2002–2003). He became group leader at the MPI in Dortmund in 2004 and Professor for Protein Chemistry at the TU München in 2007. In 2011, he became Professor and Head of the Institute of Biological Chemistry at the University of Vienna and, in 2020, founding head of the Vienna Doctoral School in Chemistry (DoSChem). He develops (bio)chemical means to generate peptides and proteins with otherwise unattainable (post‐translational) modifications*.

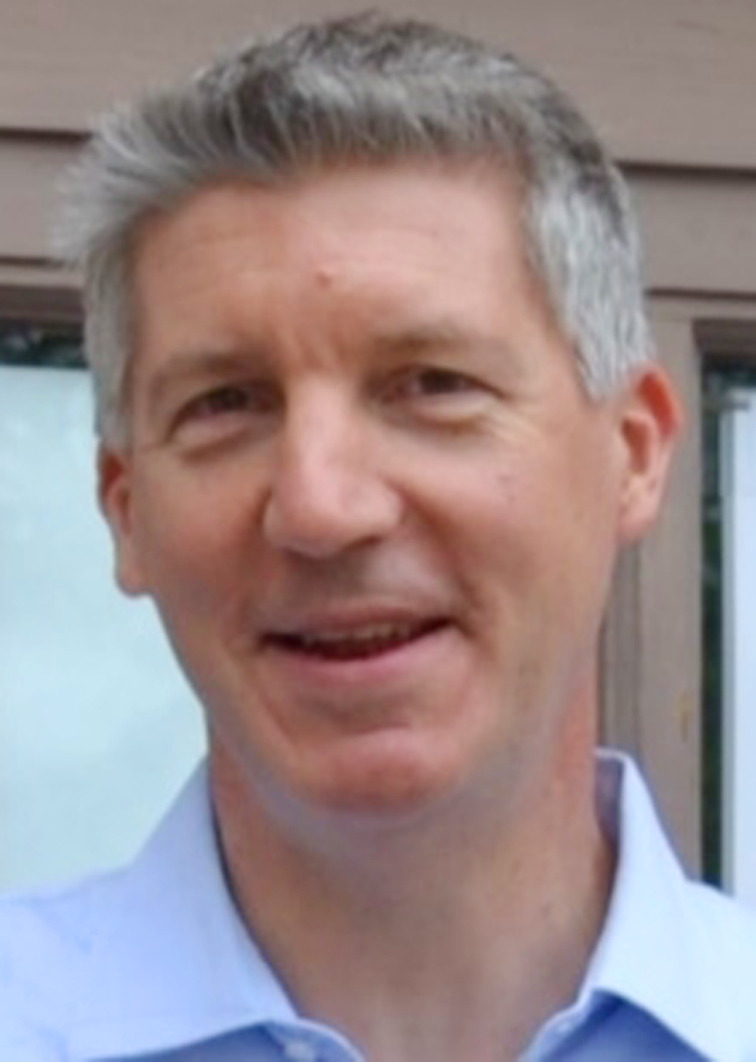

## Biographical Information

*Richard J. Payne studied Chemistry at the University of Canterbury, New Zealand and completed his PhD at the University of Cambridge in 2006 with Prof. Chris Abell. After postdoctoral research at the Scripps Research Institute with Prof. Chi‐Huey Wong (as a Lindemann Fellow), he started his independent career at the University of Sydney in 2008. Since 2015, he has been Professor of Organic Chemistry and Chemical Biology and is currently the Deputy Director of the ARC Centre of Excellence for Innovations in Peptide and Protein Science. He develops methods to access complex biomolecules to address important problems in biology and medicine*.